# Novel targeted therapies of T cell lymphomas

**DOI:** 10.1186/s13045-020-01006-w

**Published:** 2020-12-31

**Authors:** Katarzyna Iżykowska, Karolina Rassek, Dorota Korsak, Grzegorz K. Przybylski

**Affiliations:** Institute of Human Genetics, Polish Academy of Sciences, Strzeszyńska 32, 60-479 Poznań, Poland

**Keywords:** TCL, PTCL, SPTCL, Targeted therapy, HDACi, Antibodies, CART, Alki, PI3Ki

## Abstract

T cell lymphomas (TCL) comprise a heterogeneous group of non-Hodgkin lymphomas (NHL) that often present at an advanced stage at the time of diagnosis and that most commonly have an aggressive clinical course. Treatment in the front-line setting is most often cyclophosphamide, doxorubicin, vincristine, and prednisone (CHOP) or CHOP-like regimens, which are effective in B cell lymphomas, but in TCL are associated with a high failure rate and frequent relapses. Furthermore, in contrast to B cell NHL, in which substantial clinical progress has been made with the introduction of monoclonal antibodies, no comparable advances have been seen in TCL. To change this situation and improve the prognosis in TCL, new gene-targeted therapies must be developed. This is now possible due to enormous progress that has been made in the last years in the understanding of the biology and molecular pathogenesis of TCL, which enables the implementation of the research findings in clinical practice. In this review, we present new therapies and current clinical and preclinical trials on targeted treatments for TCL using histone deacetylase inhibitors (HDACi), antibodies, chimeric antigen receptor T cells (CARTs), phosphatidylinositol 3-kinase inhibitors (PI3Ki), anaplastic lymphoma kinase inhibitors (ALKi), and antibiotics, used alone or in combinations. The recent clinical success of ALKi and conjugated anti-CD30 antibody (brentuximab-vedotin) suggests that novel therapies for TCL can significantly improve outcomes when properly targeted.

## Background

**Fig. 1:**
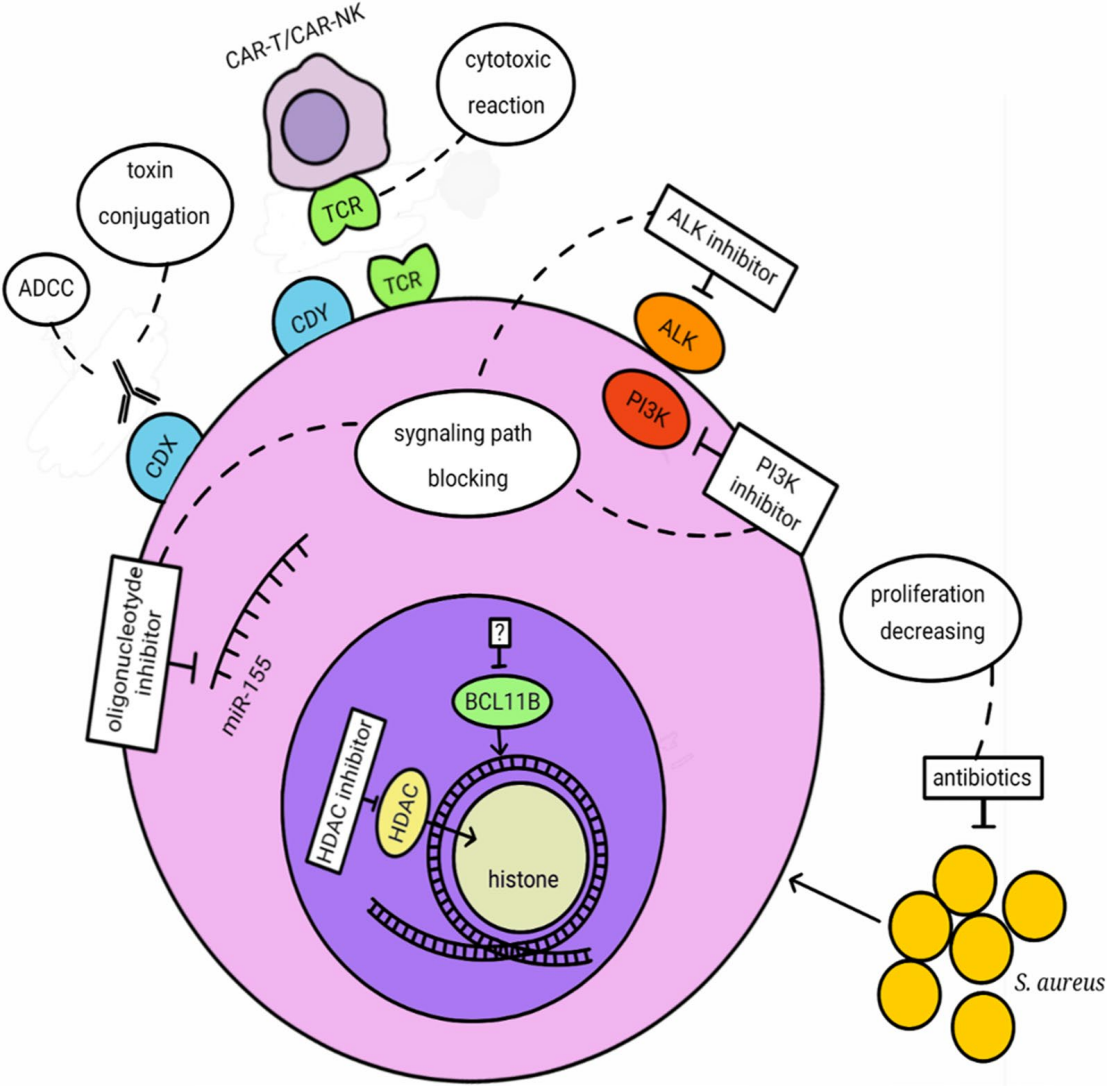
Targeted T cell lymphoma therapies mode of action. ADCC: Antibody-dependent cellular cytotoxicity, CD: cluster of differentiation antigens CDX: CD16, CD25, CD30, CD38, CD47, CD52, KIR3DL2 (CD158k), CCR4 (CD194), ICOS (CD278), CAMD1; CDY: CD4, CD5, CD7, CD30, HDAC: histone deacetylase, ALK: anaplastic lymphoma kinase, PI3K: phosphoinositide 3-kinases, BCL11B: B cell lymphoma/leukemia 11B

**Fig. 2:**
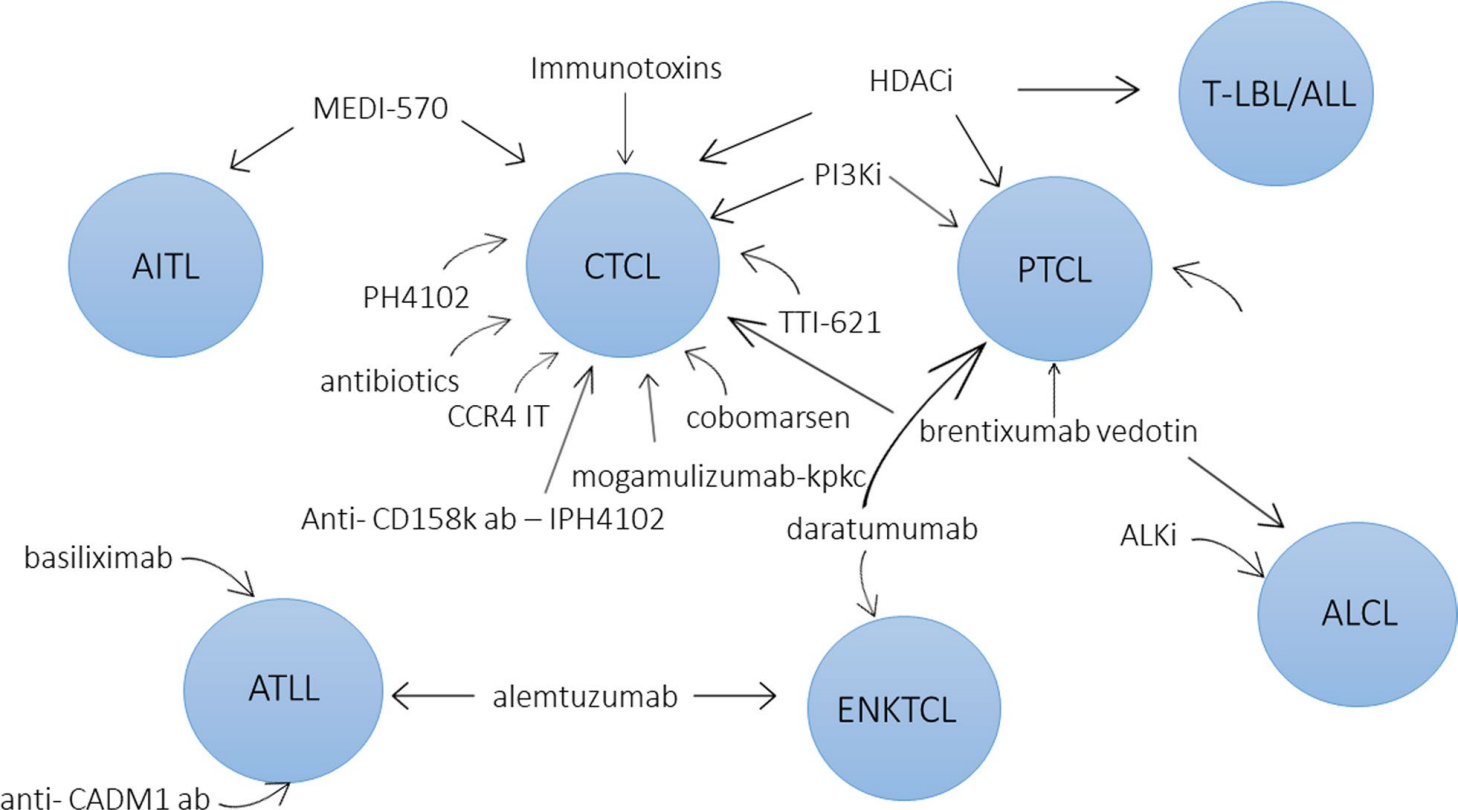
Targeted therapies in T cell lymphoma subtypes. AITL—angioimmunoblastic T cell lymphoma, CTCL—cutaneous T cell lymphoma, PTCL—peripheral T cell lymphoma, T-LBL/ ALL—T cell lymphoblastic lymphoma/T cell acute lymphoblastic leukemia, ALCL—anaplastic large-cell lymphoma, ATLL—adult T cell leukemia/lymphoma, ENKTL—extranodal NK/T cell lymphoma

T cell lymphomas (TCL) are a very heterogeneous group of lymphoid malignancies derived from mature T cells differing by localization, pathological features, and clinical presentation. TCL represent approximately 12% of all non-Hodgkin lymphomas (NHLs) and are divided into cutaneous TCL (CTCL) and peripheral TCL (PTCL), which themselves are subdivided into nodal or extranodal (systemic) types. CTCL derive from skin-homing T cells and consist of mycosis fungoides (MF), Sézary syndrome (SS), primary cutaneous CD30-positive T cell lymphoproliferative disorders: lymphomatoid papulosis (LP) and anaplastic large cell lymphoma (ALCL), cutaneous γδ TCL (CGD-TCL), cutaneous CD8-positive aggressive epidermotropic cytotoxic TCL (CD8 + AECTCL), and cutaneous CD4-positive small/medium TCL (CSM-TCL). Nodal PTCL consist of peripheral TCL not otherwise specified (PTCL-NOS), angioimmunoblastic TCL (AITK), and anaplastic large cell lymphoma (ALCL): ALK positive and ALK negative. Extranodal PTCL consist of extranodal NK/T cell lymphoma nasal type (ENKTL), enteropathy-associated TCL (EATCL), hepatosplenic TCL (HSTCL), and subcutaneous panniculitis-like TCL (SPTCL) [1]. The common features of TCL are aggressive course and poor response to therapy with the exception of ALK + ALCL. Despite the enormous progress that has been made in the twenty-first century in the treatment of hematological malignancies in the majority of TCL cases, the outcome is still unsatisfactory, and the disease remains incurable. Therefore, new targeted treatment modalities for TCL patients are currently being extensively explored. Those emerging treatments are based on histone deacetylase inhibitors (HDACi), antibodies (Ab), chimeric antigen receptor T cells (CARTs), phosphatidylinositol 3-kinase inhibitors (PI3Ki), anaplastic lymphoma kinase inhibitors (ALKi) and antibiotics, used alone, in combinations with each other, or in combination with classical chemotherapy (Figs. 1 and 2).

## Histone deacetylase inhibitors (HDACi)

Histone deacetylases (HDACs) are a group of enzymes involved in the epigenetic regulation of gene expression. They remove the acetyl group from histones and, as a result, modulate the chromatin structure and change the accessibility of transcription factors to their target DNA sequence [2]. There are four classes of HDAC: class I HDACs (HDAC1, 2, 3, 8) are ubiquitously expressed in all cell types and are localized in the nucleus, class II HDACs (HDAC 4, 5, 6, 7, 9, 10) are more tissue specific and can be localized in the nucleus and cytoplasm, class III (called sirtuins; SIRT1-7) and class IV (HDAC11). The balance between the histone acetylation by histone acetylases (HAT enzymes) and deacetylation by HDACs is often disturbed in cancer leading to altered gene expression and malignant transformation. Compounds that block HDAC, HDAC inhibitors (HDACi), were introduced to the treatment of several types of cancer, mainly in T cell lymphomas. HDACi have the capacity to increase acetylation of histones and other proteins, inducing changes in chromatin structures and the promotion of expression of tumor-suppressor genes, apoptosis, and, as a result antitumor activity [3]. HDAC inhibitors may act against all types of HDACs (pan-inhibitors) or, specifically, against some of the HDAC isoforms (HDAC isoform-selective inhibitors).

The mechanism of action of HDACi can be different; it depends on the type of cancer, the type of HDACi used, and its dose. Multiple cellular processes are activated upon HDACi treatment [2, 4]. Cell cycle arrest is induced by increased expression of genes like p21, p53, and RUNX3. Both intrinsic and extrinsic apoptotic pathways are activated, through either death receptors (TRAIL, DR5, Fas, TNF) or the activation of pro-apoptotic genes like Bid, Bad, and Bim. Autophagy is the mechanism of the anti-cancer effect of HDACi through acetylation of autophagy-related proteins, overexpression of autophagy-related genes, and also as a result of ROS production. HDACi also alters the expression of noncoding RNAs, genes involved in cell growth and differentiation (including protein kinases), major histocompatibility complex (MHC) and costimulatory molecules, and genes involved in angiogenesis and the metastatic process [2, 5]. In 2017, ATACseq analysis showed that clinical response to HDACi is strongly associated with a gain in chromatin accessibility [6]. HDACi caused distinct chromatin responses in malignant and normal CD4 + T cells, reprogramming the first ones toward normalcy. The pattern of accessible chromatin could be used to predict clinical response to HDACi [6].

## FDA-approved HDACi

### Suberoylanilide hydroxamic acid (SAHA, Vorinostat)

Vorinostat was the first drug to be approved by the FDA, since 2006, for CTCL patients with progressive, persistent, and recurrent disease on or following two systemic therapies (FDA). Vorinostat is an oral competitive inhibitor of class I/II HDAC enzymes [3]. In two phase-II trials, vorinostat 400 mg/day was safe and effective, with an overall response rate (ORR) of 24–30% in refractory advanced patients with CTCL including SS [7–9]. However, in the phase-III MAVORIC trial, vorinostat was compared with mogamulizumab in MF/SS patients and an ORR was only 5%, which was significantly lower than that of mogamulizumab (28%) [10]. The most common and serious toxicity side effects were thrombocytopenia, anemia, dehydration, nausea/vomiting, hypotension, infection, sepsis, pulmonary embolism, and deep venous thrombosis, but they were reversible upon discontinuation of the drug [11].

### Belinostat (PXD-101)

The FDA approved Belinostat in 2014 for the treatment of patients with relapsed or refractory PTCL. In a phase-II clinical trial, 24 PTCL patients and 29 CTCL patients (17MF/7SS) were administrated 1,000 mg/m^2^ intravenously on days 1–5 every 3 weeks. The ORRs were 25% for PTCL and 14% for CTCL. In total, 77% of patients experienced the following side effects: nausea, vomiting, infusion site pain, and dizziness [12]. In the pivotal phase-II BELIEF (CLN-19) study with patients with relapsed or refractory PTCL, ORR was similar: 25.8% (31 of 120), including 13 complete responses (CR) (10.8%) and 18 partial responses (PR) (15%) [13]. The most common grade 3/4 adverse events were anemia, thrombocytopenia, dyspnea, and neutropenia. In 2018, Allen and Lechowicz conducted a systemic review to assess the safety and efficacy of belinostat [14]. A safety analysis was performed on 512 patients with different relapsed malignancies from 16 different studies, and an efficacy analysis was focused on patients with relapsed PTCL (144 patients). The safety analysis showed that among all adverse events, the most common were fatigue, nausea, and vomiting, while overall grade 3/4 hematologic toxicity was low (6.4%). The efficacy analysis confirmed the ORR to be 25.7%, with 10.4% complete remissions and 15.3% partial responses.

### Romidepsin

Romidepsin was FDA-approved in 2009 for CTCL patients who have received at least one prior systemic therapy. It is administered intravenously and inhibits class I HDAC selectively. Seventy-one CTCL patients were included in a phase-II study conducted by Piekarz et al*.* in 2009 [15]. The overall response rate was 34%; four patients experienced complete responses, while partial responses were observed in 20 patients. Overall, romidepsin was well tolerated, with the main toxicities observed being fatigue, nausea, and vomiting. Another multicenter, international, pivotal study of romidepsin in refractory CTCL was conducted in 2010 [16]. Ninety-six patients were enrolled, most of whom had advanced-stage disease. The ORR was 34%, and six patients reached a complete response (CR). A clinically meaningful improvement in pruritus was observed in 43% of patients, lasting for a 6-month period. The third study, in 2011 [17], enrolled patients with CTCL and PTCL. CR was observed in 8 and PE in 9 of 45 patients, while the ORR was 38%. In both studies, drug-related adverse events were as previously described, mainly involving gastrointestinal disturbances. Romidepsin was also proven to have a good response in patients with relapsed or refractory CTCL with cutaneous tumors and/or folliculotropic disease involvement with less favorable outcomes. The ORR was 45% and 60%, respectively, and there was a significant reduction in pruritis [18]. Pruritis reduction upon romidepsin treatment was confirmed even in patients without any objective clinical response [19]. The most recent multicenter retrospective study of 53 patients with relapsed or refractory PTCL and CTCL treated with romidepsin [20] showed that the ORR and the CR rates for PTCL were 33% and 12.5%, respectively, and for CTCL, 25% and 0%, respectively. The most common grade 3/4 adverse events included hematological toxicity and infections.

### Panobinostat

The FDA approved panobinostat for the treatment of multiple myeloma (MM) in 2015. It is a pan HDACi that is orally bioavailable. To check the efficacy of this HDACi in CTCL, a phase-II study was conducted in 2012 [21]. Oral panobinostat demonstrated clinical activity in MF or SS patients regardless of prior bexarotene treatment. An ORR of 17.3% for all patients was detected, while 74% showed an improvement in the severity of their skin disease. Panobinostat was generally well tolerated, with thrombocytopenia, diarrhea, fatigue, and nausea being the most common adverse events. In 2013, another study was conducted to verify the safety, pharmacokinetics (PK), and preliminary activity of panobinostat in different hematologic malignancies, and promising single-agent activity was noted in patients with MF [22].

## HDACi in clinical trials

### Chidamide

Chidamide was approved in December 2014 by the China Food and Drug Administration (CFDA) for the treatment of relapsed or refractory PTCL. It is a selective inhibitor of HDAC1, 2, 3, and 10 and is administrated orally [23]. Seventy-nine patients with PTCL were enrolled in a first phase-II study conducted in 2015. The ORR was 28% (22 of 79) including 14% (11 of 79) with complete response/unconfirmed complete response. Most adverse events were grade 1 or 2; grade 3 and 4, which occurred in ≥ 10% of patients, were thrombocytopenia, leucopenia, and neutropenia [24, 25].

### Resminostat

Resminostat is an orally bioavailable pan-HDAC inhibitor specifically targeting class I HDACs. It was tested in clinical trials for hepatocellular carcinoma patients [26]. Now there are plans to test it in patients with advanced-stage (Stage IIB–IVB) MF or SS who have achieved disease control with systemic therapy—the RESMAIN Study (NCT02953301).

### Quisinostat

Quisinostat is a potent “second-generation” class I HDAC inhibitor with prolonged pharmacodynamic response in vivo [27]. It was shown to have the potential to inhibit cancer cell self-renewal [28]. A clinical study on Quisinostat in patients with previously treated stage Ib-IVa CTCL (NCT01486277) was conducted, though with no results available so far.

### AR-42

AR-42 (Arno Therapeutics) is an orally bioavailable, hydroxamate-tethered phenylbutyrate-derived small molecule that targets and inhibits class I and IIB HDACs. An antitumor activity of this compound in solid tumors and hematological malignancies was detected in vitro, and in 2017 the results of the phase I clinical trials were published [29]. The safety of this dug was confirmed, and the maximum tolerated dose (MTD) was established: 40 mg administered orally three times weekly, for three weeks of a 28-day cycle.

### HDACi combined with other therapies

The response to treatment with HDACi is 30%, which is still not satisfactory. Many studies have been conducted to verify the combination of HDACi with other drugs and protocols in order to achieve higher response, especially in advanced-stage patients.

Romidepsin has been tested together with both radiotherapy and chemotherapy in patients with TCL. It was shown that in advanced MF patients, localized electron beam radiation with romidepsin therapy produced a fast and durable response and that significantly lower doses of electron beam radiation effectively treated symptomatic lesions in studied patients [30]. Also, a total skin electron beam therapy (TSEBT) with romidepsin in advanced SS/MF patients was shown to be a safe option with no additional adverse events [31]. The safety and efficacy of romidepsin and other anti-cancer drugs have been investigated. Seven PTCL patients were enrolled to study the romidepsin–bendamustine combination, and among them, two achieved complete remission [32]. Eighteen patients were enrolled in a phase-I study of romidepsin and ifosfamide, carboplatin, and etoposide for the treatment of patients with relapsed or refractory PTCL [33]. The outcomes were promising. The ORR was 93%: 12 (80%) patients achieved CR and 2 patients achieved (13%) partial remission (PR); one (7%) patient achieved stable disease (SD). For the combination of romidepsin and pralatrexate, the ORR was 57% (13/23) across patients with different types of relapsed/refractory lymphomas, and 71% (10/14) in PTCL, while each drug separately usually gives an ORR of 25% and 29%, respectively [34]. The study suggested that such an approach could be an effective and safe platform for patients with PTCL; therefore, the phase-II clinical trial is now being conducted (NCT01947140).

Moreover, romidepsin could be used together with other epigenetic drugs like 5-azacytidine (AZA). Thirty-one patients with lymphoid malignancies were enrolled in a phase-I study to assess the synergistic effect between oral AZA, a hypomethylating agent, and romidepsin [35]. The overall response rates in all, non–T cell and T cell lymphoma patients were 32%, 10%, and 73%, respectively, and the complete response rates were 23%, 5%, and 55%, respectively. The combination of two drugs was more active in patients with PTCL. Among adverse effects, thrombocytopenia, neutropenia, and pleural effusion were the most severe. The clinical trial is ongoing (Phase I/IIa Study of the Oral 5-Azacitidine in Combination With the Histone Deacetylase Inhibitor Romidepsin for the Treatment of Patients With Relapsed and Refractory Lymphoid Malignancies NCT01998035). AZA was also tested with vorinostat and the combination of gemcitabine/busulfan/melphalan in patients with different types of lymphoma, including T cell types [36]. The previous observation showed that treatment with vorinostat/Gem/Bu/Mel increased the activity of methyltransferases and that further inhibition of DNA methyltransferases could enhance the cytotoxicity of this combination of drugs. The study showed higher CR rates upon azacitidine treatment combined with vorinostat/Gem/Bu/Mel especially in patients with refractory or poor prognosis relapsed HL and NH.

A phase-I clinical trial combining romidepsin and alisertib has been conducted in patients with relapsed/refractory aggressive B cell and T cell lymphoma [37]. Alisertib is a drug that inhibits aurora A kinase (AAK), a serine/threonine kinase required for cell division. The ORR for this drug alone is 27% in patients with relapsed refractory aggressive B cell and T cell NHL and even higher in patients with PTCL, between 33 and 50% [37]. However, the majority of responses are partial and short-lasting. It was shown that AAK inhibitors and HDACi have synergistic activity; HDACi create a pro-apoptotic environment and sensitize cells to AAK inhibitors. However, in a study in which romidepsin and alisertib were used together in patients with relapsed/refractory aggressive B cell and T cell lymphoma, the ORR was only 28%.

In a phase-I study, duvelisib in combination with romidepsin or bortezomib was used to determine the maximum tolerated dose in relapsed/refractory TCL patients (NCT02783625) [38]. Duvelisib (IPI-145) is an oral inhibitor of PI3K-δ and PI3K-γ. Phosphoinositide-3-kinases (PI3K) are involved in cell signaling and regulate multiple cellular functions, while PI3K-δ and PI3K-γ isoforms are crucial for T cell functioning. Inhibition of PI3K is a therapeutic strategy for PTCL and CTCL. The ORR response of duvelisib is 47% in PTCL patients, while in combination with romidepsin it was only slightly higher (50%), but well tolerated. Patients with CTCL and PTCL were also enrolled in a clinical trial to assess the combination of romidepsin with lenalidomide. Lenalidomide is an immunomodulatory agent that has antiproliferative and antineoplastic activity in malignant cells [38]. The ORR in PCTL patients treated with lenalidomide was 22–26%, while when romidepsin was used, the ORR was 50% [39]. A phase-II study in untreated PTCL with lenalidomide and romidepsin is ongoing (NCT02232516). Carfilzomib, a proteasome inhibitor used in combination with the previous two, did not increase the ORR in PCTL patients.

Multiple HDACi are also being incorporated into hematopoietic cell transplantation (HCT) approaches, in both the frontline and maintenance settings in patients with PTCLs [40]. In a recent phase-II multicenter trial, the efficacy of romidepsin was evaluated as a maintenance therapy after auto-HCT for patients with PTCL. Two patient cohorts were included: patients transplanted in CR1/PR1 (*n* = 25) and patients transplanted in CR2/PR2 or later (*n* = 7) [41]. In the first group, the estimated 2-year progression-free survival (PFS) was 49%; among this group angioimmunoblastic T cell lymphoma (AITL) patients were highly represented, with a 2-year PFS of 44%. In the second group, estimated 2-year PFS was 47%. However, PFS improvement with romidepsin maintenance was considered to be not significant, as PFS after AHCT itself is 36–45%.

The synergistic interaction between romidepsin and liposomal doxorubicin (LD) in both CTCL cell lines and primary CTCL cells was detected, and it was confirmed in the phase-I study in relapsed/refractory CTCL and PTCL [42]. This combination provided an ORR of 70% in MF and SS patients, which is a significant improvement, and only 27% in PTCL patients.

Chidamide is now being extensively tested in a preclinical and in clinical trials in combination with other drugs. Studies showed that chidamide treatment with low-dose doxorubicin exhibited a synergism effect on cell growth and apoptosis in two PTCL cell lines [43]. In a clinical trial in which chidamide and chemotherapy were used in patients with refractory or relapsed T cell acute lymphoblastic lymphoma/leukemia (T-LBL/ALL), the sensitivity of T-LBL/ALL cell to chemotherapy drugs was improved and the complete response and ORR increased [44]. A large, multicenter study was performed in China on chidamide in relapsed or refractory peripheral T cell lymphoma. In total, 383 patients were enrolled; ORR for chidamide used as a monotherapy was 39%, while when chidamide was used with chemotherapy, the ORR was 51% [24].

### HDACi—future perspective

Treatment with HDACi is promising, yet there are still several challenges. One of them is the improvement of drug delivery. The efficient oral delivery of hydrophobic molecules to target tissues is limited [45]. HDACi like vorinostat have poor solubility and permeability and, as a result, have low bioavailability. A recent study by Meka et al*.* (2018) [45] investigated the effect of the encapsulation of vorinostat within functionalized mesoporous silica nanoparticles (MSNs) on its solubility, permeability, and anti-cancer activity. All parameters were enhanced 2.6-fold and fourfold, respectively, and increased HDAC inhibition, apoptosis induction and altered gene expression in cancer cell lines were observed. To improve the selectivity of Vorinostat to cancer cells, Bhadat et al*.* (2018) generated a novel SAHA prodrug (SAHA‐OBP) that is activated in the presence of hydrogen peroxide, a reactive oxygen species (ROS) known to be overexpressed in cancer cells [46]. The analysis showed that the SAHA‐OBP prodrug is activated inside cancer cells due to the high intracellular ROS levels. The reaction between SAHA‐OBP and H_2_O_2_ produces active SAHA, which leads to the inactivation of cytosolic HDAC6, the hyperacetylation of tubulin, and, in the end, apoptosis. In another study, a synthesized SAHA-based prodrug polymer was designed, denoted as POEG-b-PSAHA. These amphiphilic polymers were shown to self-assemble into prodrug micelles and serve as nanocarriers for doxorubicin delivery [47] and increased cytotoxicity of those drugs toward tumor cells.

Another huge challenge is the resistance of cancer cells to HDACi. The mechanisms behind that resistance are still poorly known. Recently, Andrews et al*.* [48] showed differences in the acetylation levels of gene regulatory elements between HDACi-sensitive and HDACi-resistant CTCL patients. These changes were linked to the different expression of genes involved in the cell cycle, apoptosis, cytokine/chemokine signaling, and cell adhesion/migration pathways. In HDACi-resistant samples, increased acetylation was particularly significant near potential MF/SS oncogenes CCR6, CXCR4, and LAIR2 [48]. The last one was suggested to be used as an HDACi-resistant marker. What’s interesting, single-cell analysis showed that it is possible to distinguish subpopulations of SS cells that are resistant to HDACi treatment and lead to a relapse of the disease [49]. This knowledge could be useful in planning the treatment based on multiple agents targeting different populations of malignant cells. The search for more effective treatments is ongoing. Recently, bromodomain and extra-terminal motif inhibitors (BETi) are being tested in the therapy of CTCL [50, 51]. BET proteins are other epigenetic modulators, so-called readers, and BET inhibitors prevent interaction between BET proteins and acetylated histones and transcription factors. The preclinical findings on CTCL cell lines showed that epigenetic modulation with a combination of BETi and HDACi could be a beneficial therapy for CTCL. Those two drugs were shown to promote cell apoptosis and inhibit cell proliferation. Another drug that could be used in combination with HDACi is a BCl2 inhibitor, Venetoclax [52]. A subset of CTCL patients showed high sensitivity to Venetoclax; also, a synergistic effect was observed when venetoclax was combined with romidepsin and vorinostat. A synergistic effect in the induction of SS tumor cells was also detected between Vorinostat and the anticancer antibiotic Mithramycin (Plicamycin, MTR, marketed as Mithracin®), a direct inhibitor of the binding of Sp1 family factors to GC-rich promoters [53].

## Antibody-based therapies

Antibody-based therapies became one of the most important areas of treatment strategies for TCL. An unquestionable advantage of using monoclonal antibodies (mAb) compared to other strategies is their high specificity and, therefore, limited adverse effects. To date, two FDA and EMA mAb-based medicines are approved for TCL treatment; however, a number of antibody-based drugs are undergoing clinical trials, with strategies focusing on mAb not only alone but also in combination with other drugs in order to increase clinical efficacy.

## FDA-approved antibody-based drugs

### Brentuximab vedotin—anti-CD30 antibody–drug conjugate

CD30 (tumor necrosis factor receptor superfamily, member 8; TNFRSF8) is a transmembrane protein belonging to the tumor necrosis factor receptor (TNFR) superfamily. CD30 normal expression is restricted to a small subpopulation of activated B, T, and natural killer (NK) cells; however, it can be induced by a viral infection. Indeed, CD30 expression was reported on lymphocytes infected by such viruses as human immunodeficiency virus (HIV), human T-lymphotropic virus-1 (HTLV-1), or Epstein–Barr virus (EBV). The exact function of CD30 in human physiology has not yet been discovered; however, it was shown that depending on the context and target cells, CD30 expression may either suppress replication and lead to apoptosis or promote cell proliferation and survival [54]. In addition, CD30 can regulate peripheral T-lymphocyte immune responses by controlling T cell survival and downregulating cytolytic capacity as well as controlling T-helper 1 and 2 (Th1 and Th2) responses in autoimmune and inflammatory conditions by interaction with its ligand (CD30L) [55–57]. CD30 was also reported to stimulate T cells to produce such cytokines as IL-2, TNF, and IFN-γ [58]. CD30 expression is present on the tumor cells of most classical Hodgkin lymphomas as well as anaplastic large cell lymphoma (ALCL) and lymphomatoid papulomatosis (LyP). Numerous reports also identified variable CD30 expression in other lymphoproliferative disorders, such as PTCL, MF, SS, ATCLL or ENKTL [59–65]. Due to the limited CD30 expression on normal cells and the relative overexpression in certain tumor types, CD30 represents an important target for the immunotherapy of hematological malignancies.

Brentuximab vedotin is an antibody–drug conjugate (ADC) combining CD30 mAb with the microtubule inhibitor monomethylauristatin E. After ligation of ADC with CD30 on the surface of cancer cells, monomethylauristatin E binds to tubulin and disrupts the microtubule network in the cell, resulting in cell cycle arrest and apoptosis [66, 67]. Brentuximab vedotin has three main advantages. It can distinguish between normal and malignant cells and, therefore, has less toxicity in vivo. What’s more, due to monomethylauristatin E conjugation to the mAb, it remains relatively stable in the circulation, resulting in higher cytotoxicity. Finally, monomethylauristatin E released to the tumor microenvironment can kill surrounding non-targeted CD30 + malignant cells as well as non-malignant cells that may have protumor effects [68]. Brentuximab vedotin has so far been approved for the treatment of CD30 + lymphoproliferative disorders such as classical Hodgkin lymphoma, systemic and primary cutaneous ALCL, MF, AITL and PTCL = NOS.

Recently, phase-3 trial ECHELON-2 (NCT01777152) concerning the use of brentuximab vedotin in previously untreated CD30 + patients with PTCL has been initiated [69]. ECHELON-2 trial was conducted to compare the efficacy and safety of the chemotherapy regimen of CHOP (cyclophosphamide, doxorubicin, vincristine and prednisone) versus a combination of CHOP together with brentuximab vedotin (A + CHP). The results showed that the addition of brentuximab vedotin to CHP resulted in higher rates of PFS and overall survival (OS) of patients with median PFS of 48.2 months in comparison with 20.8 months in the CHOP group (p = 0.011). What’s more, the addition of brentuximab vedotin did not change the incidence and severity of adverse events such as febrile neutropenia (18% of patients in the A + CHP group and 15% in the CHOP group) and peripheral neuropathy (52% in the A + CHP group and 55% in the CHOP group). Therefore, ECHELON-2 trial results are considered to be potentially practice-changing and indicate the potential use of A + CHP treatment in CD30 + PTCL patients.

### Mogamulizumab—anti-CCR4 ab

C–C motif chemokine receptor 4 (CCR4) is a seven-transmembrane G-protein-coupled receptor expressed on Tregs, type 2 helper T cells (Th2), memory T cells and cutaneous lymphocyte antigen-positive skin-homing T cells [70]. CCR4 expression present on Th2 and Tregs induces homing of these leukocytes to sites of inflammation. Tregs play a crucial role in maintaining immune balance; however, in malignancies, Tregs attenuate the host's anti-tumor immunity and provide a favorable environment for tumor growth [71]. Elevated CCR4 expression was also reported in patients with aggressive PTCL, especially in ATLL or CTCL; therefore, CCR4 seems to be a promising therapeutic target for T cell malignancies [72].

Mogamulizumab-kpkc is a mAb directed against the CCR4 receptor, which increases antibody-dependent cellular cytotoxicity (ADCC) in CCR4 + malignant T cells. In addition to directly targeting malignant T cells, mogamulizumab depletes CCR4 + Tregs, which is an important therapeutic target in many human malignancies due to their role in suppressing the host anti-tumor immunity [73]. In 2018, the FDA-approved mogamulizumab-kpkc was approved in 2018 by FDA for the treatment of refractory MF and SS after at least one prior systemic therapy.

## mAb in clinical trials

### Anti-KIR3DL2 (CD158k) ab–IPH4102

KIR3DL2, also known as CD158k, belongs to the family of killer cell immunoglobulin-like receptors (KIRs) normally detected on a minor NK cell subset and on rare CD3 + CD8 + T cells. Although it was shown that KIR3DL2 ligation on NK cells inhibits their production of IFN-γ and cytotoxic function, KIR3DL2 function on T cells is less clear [74]. To date, numerous studies have identified elevated KIR2DL2 expression in transformed MF, pcALCL and SS [75–78]. In Sézary patients, KIR3DL2 was shown to act as an inhibitory co-receptor that promotes resistance to activation-induced cell death by its ability to down-modulate CD3-dependent early signaling events [79]. In addition, as the percentage of KIR3DL2 + peripheral mononuclear cells strongly correlates with the percentage of atypical circulating SS cells, KIR3DL2 is considered to be a diagnostic and prognostic marker for this disease [80]. Due to the limited expression of KIR3DL2 on normal immune cells and its high expression on malignant T cells, novel anti-KIR3DL2 therapeutic strategies have been proposed.

IPH4102 is an anti-KIR3DL2 mAb that was shown to deplete KIR3DL2 + cells through antibody-dependent phagocytosis and cell cytotoxicity [81]. The anti-tumor activity of IPH4102 was firstly shown in mouse xenograft models and further confirmed in an ex vivo model using the primary cells of SS patients, where it reduced tumor growth and improved cell survival. Those encouraging preliminary data resulted in a phase-I study in patients with relapsed or refractory CTCL, especially those with SS (NCT02593045) [82]. IPH4102 was associated with a favorable safety profile, high frequency of sustained global response and improvement of life quality, with peripheral edema and fatigue as the most common adverse effects. Overall response was achieved in 16 of 44 patients (36·4%), and of those, 15 were observed in 35 patients with SS (43%). Currently, phase II of IPH4102 is ongoing to confirm IPH4102 activity alone or in combination with chemotherapy in SS patients and other TCL subtypes that express KIR3DL2 (TELLOMAK, NCT03902184)**.**

### Anti-CD38 ab–daratumumab

CD38 is a type-II multifunctional transmembrane glycoprotein, with both ectoenzymatic and receptor functions, that can be found on the surface of terminally differentiated plasma cells, as well as T cells, NK cells and on myeloid cells at different stages of development [83, 84]. Its expression was reported in NK/T cell lymphomas and recently in AITL and PTCL-NOS [85, 86]. Because of its function in the regulation and immunomodulation of metabolic pathways and also abnormal expression in hematologic malignancies that correlate with cell proliferation and disease progression, CD38 seems to be an attractive target for antibody-based therapies [83].

Daratumumab is the first-class mAb to target CD38 + myeloid-derived suppressor cells (MDSC) and regulatory T cells currently approved as a therapy for MM [87, 88]. Daratumumab targets CD38 causing tumor cell death through such mechanisms as antibody-dependent cellular phagocytosis (ADCP) or antibody-dependent cell-mediated cytotoxicity (ADCC) [89, 90]. After the promising outcome of the phase-II study (NCT02927925) involving daratumumab treatment in relapsed or refractory natural killer/T cell lymphomas with overall response rate (ORR: 25%, 8/32 patients), a new trial is currently ongoing [91]. A phase-II study is now underway to evaluate the efficacy of daratumumab in combination with gemcitabine, cisplatin and dexamethasone in patients with PTCL-NOS, AITL and other nodal lymphomas of T follicular helper cells (TFH cells) origin. In this study, refractory/relapsed patients were included, after at least one, but no more than two previous therapeutic approaches (NCT04251065).

### Anti-CD25 (IL-2Rα) ab—basiliximab and camidanlumab

CD25 is an alpha subunit of interleukin-2 receptor (IL-2R) expressed mainly on the surface of the mature T cell membrane, triple-negative thymocytes, B cells and bone marrow pre-B cells [92]. In normal conditions, CD25 can induce the affinity of IL-2R and IL-2 as well as induce CD4 + CD25 + Treg proliferation and differentiation [93]. High level of CD25 was reported in many hematological malignancies, including AITL and ALCL [94]. It was shown that upregulated CD25 expression in T cells promotes lymphomagenesis and drug resistance. Furthermore, an elevated serum level of soluble IL-2 receptor α (IL-2Rα) in NKTCL patients was significantly correlated with response to treatment and survival rate [92, 95]. It is also hypothesized that CD25 can be present on leukemic stem cells and induce oncogenic signaling pathways [92].

Basiliximab is a chimeric mAb that binds the α chain of CD25, leading to the competitive inhibition of T cell proliferation and, as a consequence, the inhibition of T cell activation [96]. After a successful phase-I clinical trial using ^131^Iodine-labeled basiliximab that showed complete or partial responses in patients with CD25 + lymphomas, follow-up trials are currently underway [97]. Yttrium Y 90 basiliximab together with standard combination chemotherapy (carmustine, cytarabine, etoposide, and melphalan (BEAM)) is currently being evaluated in a phase-I study in patients with mature TCL (NCT02342782). Due to the previous results indicating that an elevated level of CD25 correlates with chemotherapy resistance and that CD25-mediated resistance can be reversed by targeting CD25, in the phase-II study, a combination of chemotherapeutic pegaspargase and basiliximab is being investigated in the treatment of relapsed or refractory NK and T cell lymphomas (NCT04337593).

Another antibody–drug conjugate targeting CD25 currently under evaluation is camidanlumab tesirine (ADCT-301). This antibody is conjugated to cytotoxic pyrrolobenzodiazepine (PBD) dimer, which causes cell death upon cross-linking specific sites of the DNA and blocking DNA replication [98]. Recently, a phase-I trial examining the safety, tolerability and pharmacokinetics of ADCT-301 in patients with relapsed or refractory HL and NHL patients ended (NCT02432235) with the phase-II study is still ongoing (NCT04052997).

### Anti-CD47 ab—TTI-621 (SIRPαFc)

CD47 (also known as the integrin-associated protein IAP) is a transmembrane protein that belongs to the immunoglobulin superfamily. CD47 binds to several different proteins, but especially to signal regulatory protein alpha (SIRPα). CD47-SIRPα interactions are involved in many cellular processes, including proliferation, apoptosis and immune response as well as the inhibition of macrophage phagocytosis, thereby allowing cancer cells to escape immune surveillance [99, 100]. Overexpression of CD47 was reported in many hematologic malignancies, including CTCL, where it seems to be correlated with a more aggressive course and a worse clinical outcome [101, 102]. Therapies inhibiting CD47-SIRPα interaction are expected to work in two ways: firstly, by activation of adaptive immunity, resulting in cytotoxic anti-tumor responses, and secondly, by activation of innate immunity, therefore promoting cancer cells destruction by macrophages [103].

TTI-621 (SIRPa-IgG1 Fc) is a novel immune checkpoint inhibitor that blocks CD47 and prevents it from delivering an inhibitory signal to macrophages, therefore allowing them to phagocytose malignant cells [102]. The phase-I trial of TTI-621 treatment provided a promising outcome for further studies. Out of nine patients with MF or SS, one achieved CR and five additional patients experienced decreases in tumor size and/or a decreased number of circulating Sézary cells (NCT02890368) [104]. Currently, another phase-I trial of TTI-621 alone or in combination with other anti-cancer drugs (rituximab or nivolumab), in subjects with relapsed or refractory hematologic malignancies and selected solid tumors, is ongoing (NCT02663518).

In addition, another anti-CD47 mAb drug phase-I study is being conducted to evaluate the safety, tolerability, and initial efficacy of IBI188 injection in patients with advanced malignant tumors and lymphomas (NCT03763149).

### Anti-ICOS (CD278) ab—MEDI-570

Inducible T cell co-stimulator (ICOS, cluster of differentiation (CD278)) is a co-stimulatory molecule minimally expressed on naïve T cells and increasingly expressed on both activated CD4 + T cells and follicular helper T cells. It is suspected that ICOS may play an important role in the production of IL-2, IL-4, IL-5, and IFNγ from recently activated T cells as well as contribute to T cell-dependent B help in vivo [105]. While in healthy humans, expression of ICOS can be detected in 5–20% of circulating peripheral blood CD4 + T cells, studies showed that ICOS expression increases in patients with autoimmune diseases and is connected to increased pro-inflammatory cytokines expression [106, 107]. In addition, high ICOS expression on Tregs-infiltrating tumors is supposed to be associated with a poor prognosis [108].

MEDI-570 is an IgG1κ mAb that attaches to the ligand-binding domain of ICOS expressed on tumor-infiltrating CD4 + T cells, therefore preventing the interaction between ICOS + T cells and plasmacytoid dendritic cells (pDCs). This interaction leads to Treg-mediated immune suppression inhibition and the enhancement of the cytotoxic T-lymphocyte (CTL)-mediated immune anti-tumor response [109].

MEDI-570 was initially designed as a therapy for autoimmune diseases. However, a currently ongoing phase-I study will evaluate the side effects and best dose of MEDI-570 in patients with PTCL follicular variant or AITL that relapsed or did not respond to previous treatment (NCT02520791).

### Anti-CD52 ab—alemtuzumab

CD52, also known as CAMPATH-1 antigen, is a small glycoprotein expressed on the surface of mature lymphocytes, monocytes, and dendritic cells. The exact function of CD52 remains to be elucidated; however, it was shown that CD52 signal transduction leads to lymphocyte proliferation and production of TNF-α, IFNγ, and IL-6 [110]. In addition, studies demonstrated that CD52 can act as a co-stimulatory molecule inducing regulatory CD4 + T cells [111].

Alemtuzumab is a humanized anti-CD52 mAb that depletes T and B lymphocytes through mechanisms such as induction of apoptosis, antibody-dependent cellular cytotoxicity (ADCC), and complement-dependent cytotoxicity (CDC) of cells [112–117]. For now, alemtuzumab has been approved for the treatment of B cell chronic lymphocytic leukemia and relapsing forms of multiple sclerosis (MS). Alemtuzumab was previously proposed as a treatment for heavily pretreated and refractory PTCL, where, although achieving a promising overall response rate (36%), the treatment was associated with significant hematologic toxicity and infectious complications [118]. Alemtuzumab also showed promising clinical outcome and an acceptable safety profile in patients with advanced MF and SS; however, therapy was also associated with such adverse effects as cytomegalovirus (CMV) reactivation, fatal mycobacterium pneumonia, or cardiac toxicity [119, 120]. Trials were also conducted to study the effect of the combination of alemtuzumab and the chemotherapeutic regimen CHOP (cyclophosphamide, doxorubicin, vincristine, and prednisone) combination in order to improve the outcome of the treatment in PTCL and aggressive T and NK cell lymphomas; however, the addition of alemtuzumab increased the risk of infection and the toxicity of the treatment [121–123]. Attempts to improve alemtuzumab treatment outcomes are currently underway. A phase-II trial is now ongoing to determine the toxicity of alemtuzumab (Campath-1H) in combination with etoposide, prednisone, vincristine, cyclophosphamide, and doxorubicin (EPOCH) chemotherapy in non-Hodgkin's T and NK cell lymphomas (NCT00069238). The follow-up phase-I trial is now investigating the safety, toxicity profile, and maximum tolerated dose of recombinant human interleukin 15 (IL-15) in combination with standard IV alemtuzumab treatment in relapsed chronic and acute ATLL patients (NCT02689453).

### Bispecific antibody targeting both CD30 and CD16A—AFM13

CD16A, a low-affinity receptor for the IgG Fc domain, belongs to the group of transmembrane proteins expressed on NK cells, macrophages, and mast cells. Upon ligation, CD16A is responsible for inducing the lysis of target cells by NK cells and ADCC [124, 125].

AFM13 is a bispecific, tetravalent chimeric antibody construct that specifically binds to CD30, found on the cancerous cells and CD16A on NK cells and macrophages. AFM13 induces NK cell-mediated and T cell-mediated cytotoxicity and, as a consequence, tumor cell lysis [126]. AFM13 was first examined in a phase-I study of patients with relapsed or refractory Hodgkin lymphoma and demonstrated promising clinical and pharmacodynamic activity (NCT01221571). The treatment with AFM13 was well tolerated, with fever and chills being the most frequent adverse effects. Out of 26 patients included in the study, 11.5% achieved partial remission and 50% achieved stable disease [126]. Recently, a phase-Ib/IIa trial was completed evaluating the biologic activity of AFM13 in patients with relapsed or refractory CD30 + CTCL patients (NCT03192202). Preliminary results of the first three dose cohorts demonstrated that AFM13 showed promising therapeutic activity as a single agent, with an objective response rate (ORR) of 50% (4/8 patients)*.* Currently, there are two ongoing clinical trials. The first one, a phase-I study, is now examining the side effects and the best dose of AFM13 as monotherapy or modified umbilical cord NK cells combined with AFM13 in patients with CD30 + recurrent/ refractory Hodgkin lymphoma or non-Hodgkin treatment (NCT04074746). The second one is a phase-II trial to evaluate the antitumor activity and safety of AFM13 in patients with CD30 + PTCL or tMF (NCT04101331).

### Anti-CADM1 ab

Cell adhesion molecule 1 (CADM1/TSLC1) is normally involved in cell adhesion, proliferation, and differentiation [127]. CADM1 is a well-known tumor-suppressor gene in human malignancies such as liver, prostate, or pancreatic cancer [128]. However, studies showed that in the case of ATL patients, CADM1 overexpression is involved in the attachment of ATL cells to vascular endothelial cells and therefore plays a role in oncogenesis [129]. CADM1 expression was also associated with tumor growth and organ infiltration of ATL cells [130]. Therefore, is seems that CAMD1 function in malignancies depends on the origin of the cell in which it is expressed. CADM1 was reported as being a diagnostic marker for ATL; however, a recent study suggests that it can also be useful for differentiating between MF and inflammatory skin disorders [131, 132]*.*

A recent study investigated the potential of anti-CADM1 antibodies in ATLL in a mouse xenograft model. Out of all examined antibodies, one clone, 103–189, showed weak but significant antibody-dependent cellular cytotoxic activity and effectively inhibited the interaction between endothelial cells and CADM1-positive ATLL cells. In addition, treatment with the 103–189 clone remarkably suppressed the organ invasion of mouse T cell lymphoma CADM1-positive cells in a mouse xenograft model, resulting in an improved survival rate of mice [133]. Results from this preliminary study suggest that further studies should be implemented to investigate the efficacy of a combination of anti-CADM1 antibodies and chemotherapy drugs in the treatment of ATLL.

## Chimeric antigen receptor T cells (carts) immunotherapy

CAR molecules are created by combining the variable regions (Fv) of an antibody with the constant regions of the T cell receptor (TCR) chains. These molecules may be grafted into immune cells to create a tumor-specific treatment. The process of creating such a biologic drug requires choosing the proper target antigen, obtaining the cells from patients or cell line, transduction, culture expansion and infusing a sufficient number of effective and cancer-specific CAR-T cells (CARTs) or CAR-NK cells (CARNKs). The therapeutic mechanism is based on two natural functions of TCRs: antigen-binding and T cell activating [134, 135]. Cellular engineering and culturing of autologous patient T lymphocytes for their infusion have brought about a durable clinical response in cancers that had been treatment refractory by this time [136]. The use of modified immune cells encounters some natural obstacles, resulting from pathways that cancer cells use to avoid an immune response. They include inhibition of immune checkpoints (e.g., production of programmed cell death ligand 1 (PD-L1), changes in G1-regulating protein expression and changes in the metabolic environment through the secretion of suppressor factors like interleukin-10 (IL-10) and recruiting regulatory T cells). There are some strategies to revert the exhaustion of CAR-Ts, like replacement, reprogramming, and restoration of senescent cells [137].

Another issue encountered is the management of CARTs toxicities, of which the most serious are cytokine release syndrome (CRS) and neurologic toxicity, though the end-organ and hematologic toxicities are in most cases reversible [138]. CART therapy has proven to be effective in the treatment of B cell malignancies; therefore, a similar approach for treating T cell lymphomas seems to be natural next step. Finding a proper target antigen is challenging as most of them are the same for malignant and normal cells. Other important problems are fratricide and purity of harvested autologous T lymphocytes, as both malignancy and CART product recruit from the same cell population [139].

## CARTs in preclinical and clinical trials

### Anti-CD7 CARTs

CD7 expression is limited to T cells and starts appearing in the early state of lymphocyte differentiation, which makes it a great target for the treatment of T cell malignancies. It is highly expressed not only on the T-ALL blasts and about 30% of AML blasts but also in most normal T cells. CD7 CART are targeted by themselves as they also express CD7 antigens. This antigen may be removed from T cells without jeopardizing its immunocompetence. Png et al*.* applied a new approach to this problem by using a protein expression blocker (PEBL) based on an anti-CD7 single-chain variable fragment coupled with an intracellular retention domain. This was found to be an easy and effective way to obtain virtually instant abrogation of CD7 expression and to avert the fratricide effect. Cell lines and patient-derived xenograft (PDX) models have provided data confirming robust and specific cytotoxicity against investigated T cell malignancies, including ETP-ALL which is one of the most aggressive types. The authors suggest minimizing MRD before allogeneic hematopoietic stem cell transplantation, as the use of anti-CD7 cells, leads to the depletion of normal T lymphocytes and immunodeficiency [140]. Another way to manage the issue of sharing CD7 between CARTs and malignant cells was proposed by Cooper et al*.* They deleted CD7 along with the T cell receptor alpha chain (TRAC) using CRISPR/Cas9 and generated CARTs targeting CD7 (UCART7). Removal of TRAC blocks TCR-mediated signaling, permitting the safe use of allogeneic T cells and allowing for the creation of an “off-the-shelf” product with no risk of contamination of autologous T lymphocytes with malignant cells. The cells obtained efficiently killed human T-ALL cell lines and patient-derived primary T-ALL in vitro and in vivo in the murine model, without resulting in xenogeneic GvHD [141]. Obtaining an adequate number of autologous T cells without malignant cell contamination is technically difficult. Therefore, You et al*.* have investigated the possibility of using the NK-92MI cell line to modify its TCR against the CD7 antigen. Cell lines and a mouse model were used in those experiments. The use of CAR-NK cells in the animal model has shown no significant toxicity, but a reduction in tumor burden and tumor growth was followed by significant survival prolongation. Compared with CAR-T cells, CAR-NK cells demonstrate three main advantages: direct killing of cancer cells by toxic granules, smaller cytokine release which brings a lower risk of CRS, and, last but not least, the possibility of “off-the-shelf’ product development [135].

### Anti-CD4 CARTs

Because CD4 is expressed on helper T lymphocytes and CD4-positive malignancies, a preclinical study on NSG mice with T-ALL tumors was conducted to investigate the possibility of using CARTs directed against this antigen [142]. The study showed a longer survival time and about 80% more effective tumor reduction in comparison with naïve T cells treatment. Alemtuzumab was used and proved to be efficient as a safety mechanism to eliminate CARTs after treatment. Following these results, clinical studies were planned. Currently, 3 recruiting clinical phase-I studies are investigating the clinical response, safety, and pharmacokinetics of using CD4-specific CARTs in patients with CD4 + T cell leukemias/lymphomas (NCT04162340, NCT04219319, NCT03829540). Because CARTs express CD4 themselves, similar to targeting CD7, NK-92 cells were tested in vitro and in vivo in a mouse model to kill CD4-positive malignant cells [143]. In vitro PTCL cell lines derived from both adult and pediatric primary cells were sensitive to CD4-CARNK treatment. A xenograft mouse model also showed that anti-CD4 CAR-NK cells were more effective compared with vector control NK-92.

### Anti-CD5 CARTs

CD5 is a negative TCR regulator present not only on normal T lymphocytes and thymocytes but also on T-ALL and many PTCL subtype cells. Raikar et al*.* have tested NK-cell and CD5-depleted Jurkat T cell lines as CAR carriers in the treatment of T cell malignancies in vitro and in a xenograft T cell leukemia mice model [144]. Both strategies were found to be effective. The lack of significant immunosuppression and the in vitro*/*in vivo efficacy of anti-CD5 CARTs/CARNKs open the gate to investigating further possibilities regarding the adoption of cell therapy utilizing this antigen for T cell leukemias and lymphomas. A phase-I clinical trial took this approach to 10 patients with T cell malignancies expressing CD5 on at least 50% of malignant cells. Nine patients were evaluated; a response was noted in 4 and CRS in 3 of them [145].

### Anti-CD30 CARTs

CD30 is a transmembrane receptor and a member of the tumor necrosis factor (TNF) receptor superfamily. It is expressed on a small subset of activated normal (non-malignant) lymphocytes and is a common surface molecule for ALCL. It is also expressed in a subset of MF, PTCL, and ATLL [54]. However, the risk of premature elimination of T or B cells during virus responses was taken into consideration, though in an ex vivo study, the anti-CD30 CAR-T cells did not impair cellular immune responses [146, 147]. This suggests that the expression of the CD30 molecule on the memory T cells is not sufficient for being recognized and killed by anti-CD30 CARTs [148].

CD30 expression on hematopoietic stem and progenitor cells (HSPCs) during activation may lead to disorders of hematopoiesis including bone marrow aplasia. However, HSPCs compared to CTCL cells show resistance against CAR-Ts-driven lysis and when co-cultured with anti-CD30 CAR-Ts formed almost normal myeloid colony formation [149, 150]. Moreover, in humanized mice during HSPCs reconstitution autologous CD30-directed CAR-T cells do not impair human peripheral T and B cells, which allows us to presume low bone marrow toxicity of anti-CD30 CAR-Ts [150].

The presence of increased levels of soluble CD30 in the plasma of patients with HL and ALCL could raise concerns about competitive CAR binding; however, in vitro studies demonstrated that it did not negatively impact the activity of anti-CD30 CAR-Ts [146, 150].

### Anti-TCR

Targeting TCR itself seems to also be a promising approach. Beta-chain regions are coded by two different genes, TRBC1 and TRBC2. In healthy adults, T cells express one of the two in about equal numbers but malignancy develops from only one type [149]. In this situation, targeting one of them would keep the other population intact, thereby ensuring the proper immunity of patients and preventing the fratricide of CARTs. TRBC1- or TRBC2-targeting CARTs are in preclinical studies [151] and in phase-I/II ongoing clinical trials in patients with relapsed or refractory TRBC1 positive TCL (AUTO4) (NCT03590574).

## Other approaches

### Immunotoxins

Immunotoxins are hybrid molecules containing a biologic toxin chemically conjugated to monoclonal antibody, cytokine, or growth factor that binds specifically to target cells [152]. Immunotoxins are predicted to be more efficient than mAb in target tissues such as bone marrow and skin, where mAb have poor therapeutic functions due to a lack of accessory cells from the innate immune system to initiate antibody-dependent cellular phagocytosis, antibody-dependent cellular cytotoxicity, or complement-dependent cytotoxicity [153]. To date, the FDA has approved only one immunotoxin for the treatment of T cell hematologic malignancy. With overall response rates between 30 and 50%, denileukin diftitox (anti-CD25; Ontak**®**) was approved in 1999 for the treatment of persistent or relapsed CD25-positive CTCL [154, 155]. The drug was used until 2014, when, due to production issues related to E. coli expression and purification, its marketing was discontinued. Denileukin diftitox was composed of two components: a full-length sequence of IL-2, which could bind to the IL-2 receptor on T cells, and a modified cytotoxic diphtheria toxin amino acid chain [156].

Recently, Wang et al*.* once more investigated the potential of IL2 fusion toxin. The group compared the efficacy of IL-2 fusion toxin with developed anti-human CCR4 immunotoxin (CCR4 IT) and demonstrated that CCR4 IT showed greater tumor response in a CD25 + CCR4 + CTCL mouse model than IL-2 fusion toxin. What’s more, the group constructed an IL2-CCR4 bispecific IT and showed that it was significantly more effective than either IL2 fusion toxin or CCR4 IT alone, therefore presenting a novel, promising targeted therapeutic drug candidate for the treatment of refractory and relapsed CTCL patients [153].

Currently, E7777, a new version of Ontak**®** with improved purity and a high percentage of active monomer is being tested in patients with persistent or recurrent CTCL. In a phase-I study carried out in Japanese patients, E7777 showed an objective response rate of 38%, with preliminary but clinically meaningful antitumor activity observed [157]. The phase-III clinical trial is now underway (NCT01871727).

### miR-155 inhibitor  (cobomarsen)

MicroRNAs (miRNAs) are small, 21–22-nucleotide (nt) noncoding RNAs that function as a posttranscriptional regulators of protein expression in normal and pathological cellular processes [158]. miR-155 plays a role in the immune response, lymphocyte development, function and differentiation [159, 160]. In addition, an elevated level of miR-155 is associated with genomic instability of malignant cells, sustained cell proliferation and survival [161]. Increased expression of miR-155 was shown in many solid tumors and hematological malignancies, including NKTCL and CTCL [162, 163]. miR-155, a microRNA associated with poor prognosis in lymphoma and leukemia, has been implicated in the progression of MF [153], the most common form of CTCL.

Cobomarsen is a synthetic locked oligonucleotide inhibitor of miR‐155 that was shown to inhibit cell proliferation and induce cell apoptosis in MF and HTLV‐1 + CTCL cells [164]. Cobomarsen is currently being tested in three clinical trials. A phase-I study is being conducted to establish the safety, tolerability, pharmacokinetics, and potential efficacy of the tested drug in patients with certain lymphomas and leukemias, including CTCL (NCT02580552).

An ongoing phase-II trial is focused on comparing the effects of the efficacy and safety of cobomarsen to vorinostat, a drug that has already been approved for the treatment of CTCL (SOLAR, NCT03713320). Another phase-II study, which is a follow-up to the SOLAR study, focuses on patients who have confirmed disease progression following treatment with vorinostat and will reveal the tolerability and safety of cobomarsen based on the potential side effects (PRISM, NCT03837457).

## Phosphoinositide 3-kinase δ/γ inhibitors(pi3ki)

Phosphatidylinositol 3-kinase (PI3K) is a lipid kinase involved in intracellular signal transduction. Four catalytic subunits of PI3K exist in human cells (α, β, δ, and γ) [165]. The PI3K-δ and PI3K-γ isoforms are preferentially expressed in leukocytes and extensively modulate both innate and adaptive immune function [166]. Multiple pathways mediated by PI3K-δ and/or PI3K-γ contribute to the survival, proliferation, and differentiation of malignant hematopoietic cells through tumor cell-autonomous effects. At the same time, cancer cells can modulate the tumor microenvironment through juxta-, para-, and endocrine effects on non-malignant stromal and immune cells that involve PI3K signaling. Recent studies have suggested that PI3K-γ may also suppress antitumor immune responses involving innate and adaptive effector cells [167]. PI3K-γ signaling functions through C/EBPβ as a key inhibitor of phagocytosis by tumor-associated macrophages (TAMs). In this state, TAMs negatively regulate effector T and NK cells by secreting soluble immunosuppressive factors and expressing membrane-bound immune checkpoint molecules such as PD ligand 1 (PDL1). Selective inhibition of PI3K-γ in solid tumor models can induce an immunostimulatory transcriptional program and M1 macrophage phenotype that restores CD8 + T cell activation. Thus, there are at least 3 different mechanisms through which PI3K-δ,γ inhibition could be active against lymphoid malignancies. The first involves the blocking of mitogenic and survival signaling within the tumor cell (cell autonomous). The second involves the blocking of mitogenic and survival signaling induced by factors within the tumor microenvironment, including cytokines, chemokines, and juxtacrine interactions. Finally, inhibition of PI3K-δ, PI3K-γ, or both together could activate anti-lymphoma immune responses.

### PI3Ki in clinical trials

Duvelisib (IPI-145) is an oral, dual inhibitor of PI3K-δ and PI3K-γ [168]. PI3K-δ/γ inhibition may directly inhibit malignant T cell growth, making duvelisib a promising candidate for patients with PTCL or CTCL. Inhibition of either isoform may also contribute to clinical responses by modulating non-malignant immune cells. These dual effects were investigated in a TCL cohort from a phase-1, open-label study of duvelisib in patients with relapsed or refractory PTCL (*n* = 16) and CTCL (*n* = 19), along with in vitro and in vivo models of TCL (NCT01476657) [169]. The overall response rates in patients with PTCL and CTCL were 50.0% and 31.6%, respectively (P = 0.32). There were 3 complete responses, all among patients with PTCL. Activity was seen across a wide spectrum of subtypes. The most frequently observed grade 3 and 4 adverse events were transaminase increases (40% alanine aminotransferase, 17% aspartate aminotransferase), maculopapular rash (17%), and neutropenia (17%). In summary, duvelisib demonstrated promising clinical activity and an acceptable safety profile in relapsed/refractory TCL, as well as preclinical evidence of both tumor cell–autonomous and immune-mediated effects.

Tenalisib (RP6530) is a novel, highly specific, dual PI3K-δ/γ inhibitor with nano-molar potency. In the first phase-I, open-label study to evaluate the safety, pharmacokinetics, and efficacy of tenalisib in patients with relapsed/refractory hematologic malignancies, 35 patients were enrolled [170]. No dose-limiting toxicity was reported at any of the dose levels. The most common treatment-emergent adverse events irrespective of causality were asthenia and cough in 15 (43%) patients and pyrexia in 13 (37%) patients. The most frequently reported related treatment-emergent adverse events (TEAE) were diarrhea, nausea, and vomiting. Related grade 3/4 adverse events were limited to events of hypertriglyceridemia, neutropenia, and diarrhea. Of 31 patients included in the efficacy analysis, a complete response was seen in 2 (7%) patients and a partial response in 4 (13%) patients, with an overall response rate of 19% and a disease-control rate of 61%. The median duration of response was 5.7 months. Responders demonstrated a marked downregulation of phospho-AKT on C1D8. Tenalisib demonstrated acceptable safety up to 1200 mg twice a day with no dose-limiting toxicities. A consistent clinical response was seen at doses of 200 mg BID and above. Pharmacodynamics correlated well with clinical outcome. Further phase-I/II studies are being undertaken to evaluate efficacy across different histologies.

In a second study, histologically confirmed patients, with ≥ 1 prior therapy, received tenalisib orally in a 28-day cycle in doses of 200 to 800 mg twice daily in the escalation phase (*n* = 19) and 800 mg twice daily in the expansion phase (*n* = 39) [171]. The most frequently reported TEAE and related TEAE were fatigue (45%) and transaminase elevations (33%), respectively. The most frequently reported related grade ≥ 3 TEAE was transaminase elevation (21%). Two dose-limiting toxicities occurred in the 800 mg fed cohort; hence, an 800 mg fasting dose was deemed MTD. Tenalisib was absorbed rapidly with a median half-life of 2.28 h. ORR in 35 evaluable patients was 45.7% (3 CR and 13 PR), and the median duration of response was 4.9 months. Responding tumors showed a marked downregulation of CD30, IL-31, and IL-32α. With acceptable safety and promising clinical activity, tenalisib can be a potential therapeutic option for relapsed/refractory TCL. Currently, a phase-I/II combination study with romidepsin is ongoing (NCT03770000). The safety and efficacy data support the development of tenalisib as monotherapy or in combination with existing or novel targeted therapies in patients with hematological malignancies. Ongoing data from studies of tenalisib as monotherapy in indolent NHL (NCT03711578) and in combination with romidepsin in TCLs (NCT03770000) indicate that tenalisib is well tolerated. With a favorable safety profile and promising clinical activity, tenalisib holds promise as an emerging potential therapeutic option for patients with relapsed/refractory TCL.

## Anaplastic lymphoma kinase inhibitors (Alki)

Anaplastic lymphoma kinase (ALK) is a receptor tyrosine kinase belonging to the insulin receptor superfamily, sharing a high degree of homology with leukocyte tyrosine kinase (LTK) [172]. As a receptor tyrosine kinase of insulin receptor (IR) subfamily, anaplastic lymphoma kinase (ALK), has been validated to play important roles in various cancers, especially in non–small cell lung cancer (NSCLC) and anaplastic large cell lymphoma (ALCL). The presence of ALK fusion proteins and the constitutive ALK tyrosine kinase activity represent a therapeutic target in all malignancies with ALK rearrangement. Further, considering that ALK is not widely expressed in adult tissue, few toxic effects might be expected from treatment aimed at blocking ALK function. Currently, the FDA has approved five small-molecule inhibitors of ALK, including crizotinib, ceritinib, alectinib, brigatinib, and lorlatinib, against ALK + ALCL. Novel type-I1/2 and type-II ALK inhibitors with improved kinase selectivity and an enhanced capability to combat drug resistance have also been reported [173–175]. Moreover, the “proteolysis targeting chimera” (PROTAC) technique has been successfully applied in developing ALK degraders [176–178], which opened a new avenue for targeted ALK therapies.

## ALKi in clinical trials

### Crizotinib

ALCL-inclusive trials and case series of ALCL patients treated with the first-generation ALK inhibitor crizotinib have yielded remarkably positive results, particularly in the pediatric population [179, 180].

In a study by the Children’s Oncology Group (COG), 21 out of 26 pediatric patients exhibited a complete response to ALK inhibition using crizotinib as a front-line monotherapy [181]. Unfortunately, the discontinuation of crizotinib led to abrupt relapse of ALK-Positive lymphoma patients [182].

### Ceritinib

A phase 1b study of ceritinib was conducted in patients with ALK + ALCL (NCT01283516). The study showed that two of three ALK + ALCL patients treated at a dose of 750 mg/d achieved CR and 1 PR [183]. The responses were ongoing for all 3 patients, with durations of > 20 months. Two patients experienced adverse events that required ceritinib dose reductions. A recently completed phase Ib study evaluating crizotinib in ALCL demonstrated an overall response rate of 53%, with 47% of patients obtaining a complete remission [184].

Currently, several clinical trials are running on crizotinib, lorlatinib, and ceritinib (NCT03505554, NCT02419287, and NCT01979536) with promising preliminary results. Despite the preliminary successes reported for ALK kinase inhibition in ALK + ALCL, resistance mutations have been reported [185], decreasing the sensitivities of ALCL cells to various ALK inhibitors [186]. Therefore, a cocktail of ALK inhibitors, as compared to a single inhibitor, may prove to be most effective if used upfront to preempt selection for resistant clones that would lead to relapse.

### BCL11B inhibition

B cell lymphoma/leukemia 11B gene (*BCL11B*) encodes a Krüppel‐like C2H2-type zinc finger transcription factor playing an important role in T cell development. It has been shown by us and others that *BCL11B* is overexpressed in T cell neoplasms [187, 188] and that suppression of *BCL11B* using siRNA leads to massive apoptosis of malignant T cells but not normal T lymphocytes [189]. The selective Bcl11b dependence of transformed T cells makes it an attractive target for novel therapeutic strategies directed against T-ALL and TCLs. Our group is currently running experiments in an inducible *BCL11B* knock-out mouse model spontaneously developing T-ALL to determine the therapeutic effect of *BCL11B* suppression. However, to date, a specific *BCL11B* inhibitor has not been discovered.

## Antibiotic treatment

Due to the progressive immunodeficiency and skin barrier breakdown, bacterial infections constitute a major clinical problem in patients with CTCLs. Indeed, potential infectious involvement in triggering or promoting CTCLs has long been suspected, with inconsistent results from many studies [190]. One of the pathogens proposed to play a role in CTCLs pathogenesis is *Staphylococcus aureus,* as its infection was connected to disease severity [191]. It was hypothesized that staphylococcal enterotoxins (SE) provide a persistent antigen stimulus for T lymphocytes resulting in the expansion of malignant T cells [192]. Additionally, it was shown that antibiotic treatment of *S.aureus* is associated with a clinical improvement in CTCL patients [193].

Recently, Lindahl et al*.* proposed a new therapeutic strategy for CTCL patients colonized by *S.aureus* [194]. A study showed the clinical benefits of short-term, aggressive antibiotic therapy on disease activity in 8 patients with advanced-stage CTCL. Immunohistochemistry, global messenger RNA expression, and cell-signaling pathway analysis showed that antibiotic therapy resulted in decreased expression of IL-2 receptor CD25, STAT3 signaling, and cell proliferation in lesional skin. In addition, in the case of some patients, clinical improvement lasted for longer than 8 months, which proposes a novel therapeutic strategy for the treatment of advanced CTCLs.

Currently, two clinical trials are examining the efficacy of doxycycline antibiotic treatment alone or in combination with other drugs in CTCL patients. In the early phase-I study, a combined approach of doxycycline and imiquimod, a drug enhancing the host immune system to destroy cancer cells, is being investigated (NCT03116659). Additionally, a phase-II study is examining doxycycline monotherapy in patients with relapsed CTCL (NCT02341209). The results of completed and ongoing clinical trials of targeted therapies in T cell lymphomas are summarized in Tables 1 and 2, respectively.

**Table 1:** Completed clinical trials of targeted therapies in T cell lymphomas

Drug name	Drug target	TCL type	ORR	Side effects	DOR	NCT number
Vorinostat	Class I and II HDAC	Refractory, advanced CTCL	24 – 30%	Thrombocytopenia, anemia, dehydration, nausea/vomiting, hypotension, infection, sepsis, pulmonary embolism and deep venous thrombosis	106 days	-
		Progressive, or recurrent MF/SS	29.70%		≥ 185 days	NCT00091559
		Refractory CTCL	24%		83.3—105.7 days	-
		Relapsed or refractory MF/SS	5%		21.7 days	NCT01728805
Belinostat	Class I, II, and IV HDAC	PTCL/CTCL	PTCL 25%, CTCL 14%	Nausea, vomiting, infusion site pain, and dizziness	CTCL 83 days, PTCL 109 days	NCT00274651
		Relapsed or refractory PTCL	25.80%	Anemia, thrombocytopenia, dyspnea, and neutropenia	13.6 months	-
Romidepsin	Class I HDAC	CTCL	34%	Fatigue, nausea, vomiting, asthenic conditions, diarrhea, headache, ageusia, thrombocytopenia, dysgeusia, and granulocytopenia	13.7 months	NCT00020436
		Refractory CTCL	34%		15 months	NCT00106431
		CTCL/ PTCL	38%		8.9 months	NCT00007345
		Tumor stage and folliculotropic MF	Cutaneous MF 45%,folliculotropic MF 60%		15 months	NCT00106431
		Relapsed or refractory PTCL/ CTCL	Relapsed PTCL 33%	Hematological toxicity and infections	13.5 months	-
			Refractory PTCL 12.5%			
			Relapsed CTCL 25%			
			Refractory CTCL 0%			
Romidepsin + TSEBT	Class I HDAC + skin irradiation	Advanced-stage MF/SS	No data available	Nausea, fatigue, loss of appetite, erythema, and desquamation	2-28 months	-
Romidepsin + bendamustine	Class I HDAC + DNA	Relapsed/ refractory PTCL	No data available	Nausea, vomiting in most patients, thrombocytopenia, and neutropenia	9 months	-
Romidepsin + ICE	Class I HDAC + cancer cells	Relapsed or refractory PTCL	93%	No data available	15 months	NTC01590732
Romidepsin + pralatrexate	Class I HDAC + DHFR	Relapsed/refractory lymphomas	All lymphomas 57%, PTCL 71%	Nausea, fatigue, anorexia, diarrhea, and fever	4.29 months	NCT01947140
Romidepsin + AZA	Class I HDAC + DNA methylation	Non-T cell/T cell lymphomas	All lymphomas 32%	Thrombocytopenia, neutropenia, and pleural effusion	1.8 – 16.3 + months	NCT01998035
				Non-T cell lymphoma 10%		
				T cell lymphoma 73%		
Romidepsin + alisertib	Class I HDAC + AAK	Relapsed/refractory aggressive B cell and T cell lymphomas	28%	Fatigue, nausea, infection, neutropenia, anemia, and thrombocytopenia	No data available	NCT0189701
Romidepsin + lenalidomide	Class I HDAC + E3 ubiquitin ligase	PTCL/ CTCL	53%	Neutropenia, thrombocytopenia, anemia, electrolyte abnormalities	No data available	NCT01755975
Romidepsin + liposomal doxorubicin	Class I HDAC + DNA	Relapsed/refractory CTCL and PTCL	CTCL 70%	Thrombocytopenia, anemia, neutropenia, fatigue, nausea, vomiting, and anorexia	CTCL 5.1 months	NCT01902225
			PTCL 27%		PTCL 4.2 months	
Panobinostat	HDAC	MF/SS	17.30%	Thrombocytopenia, diarrhea, fatigue, and nausea	5.6 months	NCT00425555
		Various hematologic malignancies	No data available	Thrombocytopenia fatigue, neutropenia	MF 1–369 days	-
Chidamide	HDAC 1,2,3, and 10	Relapsed or refractory PTCL	28%	Thrombocytopenia, leucopenia, and neutropenia	9.9 months	-
Chidamide + chemotherapy	HDAC 1,2,3 and 10 + cancer cells	Refractory or relapsed T-LBL/ALL	71%	Febrile neutropenia, drug-induced liver failure, decreased fibrinogen, sepsis, pneumonitis, and oral mucositis	9.4 months	-
		Relapsed or refractory PTCL	Chidamide 39.06%, chidamide + chemotherapy 51.18%	Thrombocytopenia, neutropenia, anemia, and fatigue	Chidamide 148 days, chidamide + chemotherapy 169 days	-
Alisertib	AAK	Relapsed/refractory aggressive NHL	27%	Neutropenia, leukopenia, anemia, thrombocytopenia, stomatitis, and fatigue	550 days	NCT00807495
		Relapsed/refractory PTCL or tMF	PTCL 30%	Fatigue, neutropenia, anemia, and thrombocytopenia	3 months	NCT01466881
			tMF 0%			
Brentuximab vedotin + CHP	CD30 + cancer cells	CD30 + patients with PTCL	95%	Febrile neutropenia, peripheral neuropathy	No data available	NCT01777152
IPH4102	KIR3DL2	Refractory CTCL	36.40%	Peripheral edema and fatigue	13.8 months	NCT02593045
Daratumumab	CD38	Relapsed or refractory NK/T cell lymphomas	25%	Pyrexia, headache, thrombocytopenia, anemia, leukopenia, and neutropenia	55 days	NCT02927925
TTI-621	CD47	Relapsed or refractory SS/ MF	No data available	Fatigue, chills, and decreased appetite	No data available	NCT02890368
Alemtuzumab	CD52	Pretreated and refractory PTCL	36%	Shivers, chills, significant hematologic toxicity, and CMV reactivation	2–12 months	-
		MF/ SS	55%	Fever, rigors, nausea, hypotension, anemia, neutropenia, thrombocytopenia,	12 months	-
				CMV reactivation, fatal Mycobacterium pneumonia, and adverse cardiac events		
Alemtuzumab + chemotherapy	CD52+cancer cells	PTCL	72%	Infections, leukocytopenia, thrombocytopenia, anemia	No data available	-
		CD52 + aggressive T/ NK lymphomas	83.30%		No data available	-
		Aggressive TCL	55.60%		No data available	NCT00562068
AFM13	CD30/ CD16A	Relapsed or refractory CD30 + CTCL	50%	Infusion-related reaction, cellulitis,	No data available	NCT03192202
E777	IL-2 receptor	Relapsed or refractory PTCL/ CTCL	38%	Lymphopenia, thrombocytopenia, leukocytosis, anemia, neutropenia, decreased appetite, fatigue, hypoalbuminemia, and nausea	No data available	NCT1401530
Duvelisib	PI3K-δ/-γ	Relapsed or refractory PTCL/ CTCL	PTCL 50.0%	Transaminase increases, maculopapular rash, and neutropenia	PTCL 1.8–17.3 months	NCT01476657
			CTCL 31.6%,		CTCL 0.7–10.1 months	
Tenalisib	PI3K-δ/-γ	Relapsed/refractory hematologic malignancies	19%	Asthenia, cough, pyrexia, diarrhea, nausea, vomiting, hypertriglyceridemia, and neutropenia	5.7 months	-
		Relapsed/refractory PTCL/ CTCL	45.70%	Fatigue, transaminase elevations	PTCL 6.5 months	NCT02567656
					CTCL 3.8 months	
Crizotinib	ALK	Relapsed/refractory ALCL	ALCL 83%—90%	Neutrophil count decrease	No data available	NCT00939770
Ceritinib	ALK	ALCL	No data available	Diarrhea, abdominal discomfort, vomiting, fatigue, increase in transaminase levels, acute pericarditis	≥ 20 months	NCT01283516
		ALCL	53%	Diarrhea, vision disorder, nausea, vomiting, elevated transaminases, neutropenia, leukopenia	2.6 years	NCT01121588

ORR—overall response rate; DOR—duration of response; HDAC—histone deacetylase; CTCL—cutaneous T cell lymphoma; MF—mycosis fungoides; tMF—transformed mycosis fungoides; SS—Sezary syndrome; PTCL—peripheral T cell lymphoma; TCL—T cell lymphoma; ICE—ifosfamide, carboplatin, etoposide; AZA—5-azacytidine; DLBCL—diffuse large B cell lymphoma; T-LBL/ALL—T cell acute lymphoblastic lymphoma/leukemia; CMV—cytomegalovirus; AAK—aurora A kinase; CHP—versus cyclophosphamide, doxorubicin, and prednisone; CART—chimeric antigen receptor T cell; PI3K—phosphoinositide-3-kinases; ALK- anaplastic lymphoma kinase; TSEBT—total skin electron irradiation, DHFR—dihydrofolate reductase

**Table 2:** Ongoing clinical trials of targeted therapies in T cell lymphomas

NTC number	Intervention/treatment	Title	Status	Phase	Number enrolled	Reference / Study results
NCT00336063	Drug: Azacitidine Other: Laboratory Biomarker Analysis Other: Pharmacological Study Drug: Vorinostat	Vorinostat and Azacitidine in Treating Patients With Locally Recurrent or Metastatic Nasopharyngeal Cancer or Nasal Natural Killer T-Cell Lymphoma	Active, not recruiting	1	18	Not available
NCT04220008	Procedure: Allogeneic hematopoietic stem cell transplantation Drug: Busulfan Drug: Clofarabine Drug: Cyclophosphamide Drug: Gemcitabine Drug: Mycophenolate Mofetil Biological: Rituximab Drug: Tacrolimus Drug: Vorinostat	Vorinostat and Combination Chemotherapy Before Donor Stem Cell Transplantation for the Treatment of Relapsed Aggressive B-cell or T-cell Non-Hodgkin Lymphoma	Not yet recruiting	2	30	Not available
NCT02737046	Drug: Belinostat Drug: Zidovudine Drug: Interferon-Alfa-2b Drug: Pegylated Interferon-Alfa-2b	Belinostat Therapy With Zidovudine for Adult T-Cell Leukemia-Lymphoma	Recruiting	2	20	Not available
NCT03770000	Drug: Tenalisib Drug: Romidepsin	Safety and Efficacy of Tenalisib (RP6530) in Combination With Romidepsin in Patients With Relapsed/Refractory T-cell Lymphoma	Recruiting	1 2	42	[195] 15 pts (CTCL, PTCL; 1 – dose escalation n=9; 2 – dose expansion n=6) No DLT reported in the dose escalation Tenalisib 800 mg BID+ Romidepsin 14 mg/m^2^ – optimal dose for expansion cohorts AEs: nausea (33%), thrombocytopenia (33%) and fatigue (27%) ≥ Grade 3 AEs: 33% thrombocytopenia (7%), atrial fibrillation (7%), pyrexia (7%) which were (related to romidepsin), anemia (7%) neutropenia (7%), rash (7%) (related to combination) Pts from the dose escalation cohorts (n=9): CR 3, SD 4, PD 2
NCT03141203	Drug: Romidepsin Drug: Carfilzomib	Evaluation of the Combination of Romidepsin and Carfilzomib in Relapsed/Refractory Peripheral T Cell Lymphoma Patients	Active, not recruiting	1 2	50	Not available
NCT01796002	Drug: Romidepsin + CHOP Drug: CHOP	Efficacy and Safety of Romidepsin CHOP vs CHOP in Patients With Untreated Peripheral T-Cell Lymphoma	Active, not recruiting	3	421	[196] 421 pts (Ro-CHOP, *n* = 211; CHOP, *n* = 210) median follow-up of 27.5 mo: Ro-CHOP did not show a statistically significant PFS improvement vs CHOP alone Median PFS for Ro-CHOP vs CHOP: 12.0 mo vs 10.2 mo Median OS for Ro-CHOP vs CHOP was 51.8 mo vs 42.9 mo ORR of Ro-CHOP vs CHOP was 63% vs 60% with CR + CRu rates of 41% vs 37% TEAEs: occurred ≥ 40% in the Ro-CHOP or CHOP arms: anemia (67% vs 38%), nausea (55% vs 31%), thrombocytopenia (52% vs 17%), neutropenia (51% vs 37%), and vomiting (40% vs 10%) Grade 3/4 TEAEs: occurred in ≥ 30% in the Ro-CHOP or CHOP arm: thrombocytopenia (50% vs 10%), neutropenia (49% vs 33%), anemia (47% vs 17%), leukopenia (32% vs 20%) 1 grade 5 TEAEs: Ro-CHOP arm (*E. coli*sepsis), 2 in the CHOP arm (colitis and acute cholecystitis)
NCT02616965	Drug: Romidepsin Drug: Brentuximab vedotin	A Study to Assess the Feasibility of Romidepsin Combined With Brentuximab Vedotin in Cutaneous T-cell Lymphoma	Recruiting	1	27	[197] 7 pts No G4 or 5 AEs AEs grade 3: transaminitis, fever AEs: nausea (71%), vomiting (43%), gastroesophageal reflux, constipation, peripheral sensory neuropathy, anorexia, fatigue, thrombophlebitis ORR: 80% (4/5) The median change in mSWAT: a decrease of 59% The median follow-up: 6.1 mo PFS: 12 mo
NCT02341014	Drug: Carfilzomib Drug: Romidepsin Drug: Lenalidomide	Combination Therapy With Carfilzomib, Romidepsin, Lenalidomide in Patients With Relapsed or Refractory B- and T-cell Lymphomas	Active, not recruiting	1 2	31	[198] Pts: 16 TCL, 11 BCL The MTD dose for 2: romidepsin 8 mg/m^2^, lenalidomide 15 mg, carfilzomib 36 mg/m^2^ ORR for TCL and BCL: 50% Median EFS: 14.5 weeks Median time to best response: 5.7 weeks Median duration of response: 38.7 weeks Grade 3–4 toxicities > 10%: neutropenia, thrombocytopenia AEs: anemia-1, vomiting/diarrhea-1, dyspnea-1, edema-1, febrile neutropenia-1, fever-2, generalized weakness-1, heart failure-1, hypotension-1, infection-2, gastrointestinal bleed-1, and DVT-1
NCT01755975	Drug: Romidepsin Drug: Lenalidomide	Romidepsin in Combination With Lenalidomide in Adults With Relapsed or Refractory Lymphomas and Myeloma	Active, not recruiting	1 2	62	[199] 21 pts TCL (10 CTCL, 11 PTCL) ORR: 53% (10/19) ORR in PTCL: 50% (5/10, 5 PR) Responses: PTCL-NOS (3), AITL (1), T-PLL (1) ORR in CTCL: 56% (5/9, 2 CR, 3 PR) CR: transformed MF (1), Sézary syndrome (1) Median time to response: 7.3 weeks Median OS not reached Median event-free survival: 15.5 weeks AEs ≥ Grade 3 in 71%: Neutropenia (48%), thrombocytopenia (38%), anemia (33%), electrolyte abnormalities (43%)
NCT02783625	Drug: Romidepsin Drug: Bortezomib Drug: duvelisib	Trial of Duvelisib in Combination With Either Romidepsin or Bortezomib in Relapsed/Refractory T-cell Lymphomas	Recruiting	1	115	[200] (multicenter retrospective cohort study—partial data)
NCT01908777	Other: High Dose Chemotherapy with Autologous Stem Cell Transplant Followed by Maintenance Therapy with Romidepsin	A 2 Multicenter Study of High Dose Chemotherapy With Autologous Stem Cell Transplant Followed by Maintenance Therapy With Romidepsin for the Treatment of T Cell Non-Hodgkin Lymphoma	Active, not recruiting	2	47	[200] (multicenter retrospective cohort study—partial data)
NCT03547700	Drug: Romidepsin Drug: Ixazomib	Study of Ixazomib and Romidepsin in Peripheral T-cell Lymphoma (PTCL)	Active, not recruiting	1 2	11	Not available
NCT02223208	Drug: Ro-CHOEP-21 ( I) Drug: Ro-CHOEP-21 ( II)	Ro Plus CHOEP as First Line Treatment Before HSCT in Young Patients With Nodal Peripheral T-cell Lymphomas	Recruiting	1 2	110	[201] 21 pts 1 cohort - 3 pts: Ro at 12 mg/ms -no DLTs subsequent 6 cohorts: Ro at 14 mg/ms Nine DLTs were reported in 7 pts Toxicity: 35.2% (95%CI: 17.1%-56.5%) AEs grade 3-4: neutropenia (38%), thrombocytopenia (45%) Severe extra-hematological toxicities: arrhythmia (5%), gastrointestinal (14%) and infections (24%). CR: 12 (57%) PR: 1 (5%) median follow-up: 26 mo 12-mo PFS: 52% (95%CI: 29-71) 12-mo OS: 76% (95%CI: 52-89).
NCT02232516	Drug: romidepsin Drug: lenalidomide Other: laboratory biomarker analysis	Romidepsin and Lenalidomide in Treating Patients With Previously Untreated Peripheral T-Cell Lymphoma	Recruiting	2	35	Not available
NCT03278782	Biological: Pembrolizumab Drug: Romidepsin	Study of Pembrolizumab (MK-3475) in Combination With Romidepsin	Active, not recruiting	1 2	31	[202] Pts: 20 ( I: 6 pts; II: 14 pts) II-ORR: 50% median follow-up: 18 mo CR: 5 pts PR: 2 pts AEs: nusea, vomiting, fatigue
NCT01947140	Drug: Pralatrexate Drug: Romidepsin	Pralatrexate + Romidepsin in Relapsed/Refractory Lymphoid Malignancies	Recruiting	1 2	93	[34] 23 pts All: ORR 57% (13/23), CR 17% (4/23), PR 39% (9/23) TCL: ORR 71% (10/14), CR 40% (4/10), PR 60% (6/10) AEs grade 1-2: nausea (66%), fatigue (52%), anorexia (24%), diarrhea (24%), fever (24%) AEs grade 3-4: anemia (29%), oral mucositis (14%), thrombocytopenia (14%), neutropenia (10%), thrombocytopenia (14%), neutropenia (10%), sepsis (7%), fever (3%), pneumonia (3%) PFS: 3.7 mo (all), 4.4 mo (TCL) DOR: 4.29 mo recommended 2 dose: pralatrexate 25 mg/m^2^ and romidepsin 12 mg/m^2^ every 1 week
NCT04447027	Drug: Romidepsin Drug: Lenalidomide Drug: 5-azacitidine Drug: Dexamethasone	A 1 Study of Romidepsin, CC-486 (5-azacitidine), Dexamethasone, and Lenalidomide (RAdR) for Relapsed/Refractory T-cell Malignancies	Not yet recruiting	1	30	Not available
NCT03161223	Drug: Durvalumab Drug: Pralatrexate Drug: Romidepsin Drug: 5-Azacitidine	Durvalumab in Different Combinations With Pralatrexate, Romidepsin and Oral 5-Azacitidine for Lymphoma	Recruiting	1 2	148	Not available
NCT02181218	Drug: Romidepsin Drug: Gemcitabine Drug: Oxaliplatin Drug: Dexamethasone Drug: Pegfilgrastim	I Study of Romidepsin, Gemcitabine, Oxaliplatin, and Dexamethasone in Patients With Relapsed/Refractory Aggressive Lymphomas	Active, not recruiting	1	24	[200] (multicenter retrospective cohort study – partial data)
NCT01261247	Drug: panobinostat Other: laboratory biomarker analysis Genetic: western blotting Genetic: DNA analysis Other: flow cytometry Other: pharmacological study Other: immunohistochemistry staining method	Panobinostat in Treating Patients With Relapsed or Refractory Non-Hodgkin Lymphoma	Active, not recruiting	2	41	Started: 41 pts Completed: 39 pts Proportion of Confirmed Responses Defined to be a CR or PR Noted as the Objective Status: 0.21 Median Overall Survival Time: 14.9 months Median Progression-free Survival Time: 3.1 months Duration of Response:19.7 months All-cause mortality: 0/41 (0.00%) Serious AE: 34/41 (82.93%) Other AEs: 38/41 (92.68%)
NCT04296786	Drug: Sintilimab Drug: Chidamide	Sintilimab Plus Chidamide in the Treatment of Relapsed and Refractory Cutaneous T-cell Lymphoma: a Multicenter II Study	Recruiting	2	52	Not available
NCT04038411	Drug: PD-1 Antibody, chidamide, lenalidomide and etoposide	PD-1 Antibody, Chidamide, Lenalidomide and Etoposide for Relapsed or Refractory NK/T Cell Lymphoma	Recruiting	4	50	Not available
NCT03268889	Drug: Chidamide	Chidamide With CHOP Regimen for de Novo PTCL Patients (CHOP: Cyclophosphamide, Etoposide, Vincristine and Prednisone; PTCL: Peripheral T Cell Lymphoma)	Unknown	Not Applicable	39	Not available
NCT04490590	Drug: Chidamide+ Etoposide	A Clinical Trial of Chidamide Combined With Etoposide in Relapsed or Refractory NK/T-cell Lymphoma	Recruiting	4	30	Not available
NCT04040491	Drug: PD-1 blocking antibody, chidamide, lenalidomide and gemcitabine	PD-1 Antibody, Chidamide, Lenalidomide and Gemcitabine for Peripheral T-cell Lymphoma	Recruiting	4	100	Not available
NCT04329130	Drug: Chidamide, Lenalidomide	Chidamide Combination With Lenalidomide in Patients With Relapsed or Refractory Peripheral T-cell Lymphoma	Recruiting	2	44	Not available
NCT03321890	Drug: Chidamide Drug: prednisone Drug: Cyclophosphamide Drug: etoposide Drug: Methotrexate	Chidamide Combined With PECM in Relapsed or Refractory Peripheral T-cell Lymphoma (PTCL)	Recruiting	2	102	Not available
NCT02987244	Drug: Chidamide Drug: Cyclophosphamide Drug: Epirubicin Drug: Vindesine Drug: EtoposideDrug: Prednisone	Chidamide Plus CHOEP Combined With Upfront ASCT in Untreated Peripheral T-cell Lymphoma	Recruiting	1 2	100	[203] 82 pts 1b/2 toxicities: leucopenia, anemia, and neutropenia 1b/2: ORR: 68.3%, CR 43.9% 2: ORR: 73.2%, CR 47.8% CR median follow-up: 12.7 mo 1b/2 median PFS: 17.4 mo 1-year PFS: 52.9% 1-year OS: 74.5%. 2 study median PFS: 17.4 mo 1-year PFS: 53.6% 1-year OS: 76.3%
NCT02879526	Drug: C-CPT	Chidamide Combined With Cyclophosphamide, Prednisone, Thalidomide in Treatment of Fragile Patients With Relapse/Refratory Peripheral T Cell Lymphoma	Recruiting	2	45	Not available
NCT03853044	Drug: Chidamide Drug: Cyclophosphamide Drug: Doxorubicin Drug: Vincristine Drug: Prednisone	Study Evaluating the Safety and Efficacy of C-CHOP in Untreated Subjects With Angioimmunoblastic T Cell Lymphoma	Recruiting	2	23	Not available
NCT04480125	Drug: Azacitidine Drug: Chidamide	Azacitidine Combined With Chidamide in the Treatment of Newly Diagnosed PTCL Unfit for Conventional Chemotherapy	Recruiting	2	28	Not available
NCT04414969	Drug: Anti-PD-1 antibody+Peg-Asparaginase+Chidamide	Anti-PD-1 Antibody Combined With Peg-Asparaginase and Chidamide for the Early Stage of NK/T Cell Lymphoma	Recruiting	2	35	Not available
NCT03598959	Drug: tofacitinib Drug: chidamide	Tofacitinib Combined With Chidamide in R/R ENKTCL	Not yet recruiting	2	20	Not available
NCT04511351	Drug: Chidamide	Radiotherapy Combined With GDP With or Without Chidamide in Stage I/II Extranodal Nasal NK/T-cell Lymphoma	Recruiting	Not applicable	76	Not available
NCT04319601	Drug: Rituximab	Rituximab Combined With Chidamide and Lenalidomide for r/r AITL	Recruiting	Not applicable	26	Not available
NCT03617432	Drug: Chidamide Drug: Cyclophosphamide Drug: Doxorubicin Drug: Vincristine Drug: Etoposide Drug: Prednisone	Chidamide Combined With CHOPE Regimen for Peripheral T-cell Lymphoma Patients	Recruiting	2	114	Not available
NCT03820596	Drug: Sintilimab Drug: Chidamide	Sintilimab in Combination With Chidamide in Refractory and Relapsed ENKTCL	Recruiting	1 2	50	Not available
NCT03629873	Drug: Chidamide	Efficacy and Safety of Chi-BEAC Combining With Auto-HSCT to Treat Aggressive Lymphoma Subjects	Recruiting	2	69	Not available
NCT04480099	Drug: CHOP for 6 cycles Drug: CHOP+X for 6 cycles	Targeted Drug Combined With CHOP in the Treatment of Newly Diagnosed Peripheral T-cell Lymphoma	Recruiting	2	106	Not available
NCT03553238	Drug: Chidamide Drug: Dexamethasone Drug: vincristine Drug: Cyclophosphamide Drug: Idarubicin Drug: Pegaspargase Drug: Adriamycin Drug: Methotrexate Drug: 6-Mercaptopurine Drug: Etoposide Drug: Cytarabine Procedure: Bone marrow aspiration Procedure: Intrathecal injection Radiation: Radiation therapy Genetic: NGS Procedure: allogeneic hematopoietic stem cell transplantation Diagnostic Test: Flow-MRD Diagnostic Test: FISH Diagnostic Test: Flow immunophenotyping Diagnostic Test: Karyotyping	Precision Diagnosis Directing HDACi Chidamide Target Therapy for Adult ETP-ALL	Unknown status	3 4	70	[204] 24 pts ETP-ALL Chidamide: a dose of 10 mg/day AEs: fatigue, nausea, vomit, neutropenia, thrombocytopenia CR: 87% Flow MRD-negative rate: 67% 6 pts with ETP-ALL (25%, 6/24) underwent allogeneic hematopoietic stem cell transplantation (allo-HSCT) median follow-up: 20 mo (range, 7-31 mo) estimated 2-year event-free-survival (EFS): 83%
NCT03925428	Drug: Entinostat Drug: Molibresib	Testing a New Anti-cancer Drug Combination, Entinostat and GSK525762C, for Advanced and Refractory Solid Tumors and Lymphomas	withdrawn	1	0	Not available
NCT02953301	Drug: resminostat Drug: Placebo	Resminostat for Maintenance Treatment of Patients With Advanced Stage Mycosis Fungoides (MF) or Sézary Syndrome (SS)	Recruiting	2	190	Not available
NCT01486277	Drug: Quisinostat, 12 mg	A Study of the Histone Deacetylase Inhibitor (HDACi) Quisinostat (JNJ-26481585) in Patients With Previously Treated Stage Ib-IVa Cutaneous T-cell Lymphoma	Completed	2	26	[205] 26 pts I – 6 pts 8 mg dose; no CR, PR II – 20 pts 12 mg dose; 1 CR, 3 PR 6/9 pts achieved 50% reduction in mSWAT score at least once, ORR: 21,1% AEs 1-2 grade: nausea (23%), diarrhea (19%), asthenia (12%), thrombocytopenia (12%), hypertension (8%), lethargy (8%), palpitations (8%), pruritus (8%), vomiting (8%) Grade ≥3 AEs: hypertension (4%), lethargy (4%), pyrexia (4%), hyperkalaemia (4%)
NCT01998035	Drug: Romidepsin Drug: Oral 5-Azacitidine	Romidepsin Plus Oral 5-Azacitidine in Relapsed/Refractory Lymphoid Malignancies	Active, not recruiting	1 2	52	[206] 31 pts MTD: AZA 300 mg on days 1 to 14 and ROMI 14 mg/m^2^ on days 8,15 and 22 on a 35-day cycle ORR/CR: 32%/23% (all), 10%/5% (non-T-cell lymphoma), 73%/55% (T-cell lymphoma) AEs: thrombocytopenia, neutropenia, pleural effusion
NCT02783625	Drug: Romidepsin Drug: Bortezomib Drug: duvelisib	Trial of Duvelisib in Combination With Either Romidepsin or Bortezomib in Relapsed/Refractory T-cell Lymphomas	Recruiting	1	115	[200] (multicenter retrospective cohort study – partial data)
NCT02232516	Drug: romidepsin Drug: lenalidomide Other: laboratory biomarker analysis	Romidepsin and Lenalidomide in Treating Patients With Previously Untreated Peripheral T-Cell Lymphoma	Recruiting	2	35	Not available
NCT02593045	Biological: IPH4102	Study of IPH4102 in Patients With Relapsed/Refractory Cutaneous T-cell Lymphomas (CTCL)	Active, not recruiting	1	60	[82] 44 patients: 35 SS, 8 MF, 1 PTCL-NOS Dose of 750 mg AEs: Grade 1–2: peripheral edema 27%, fatigue 20% Grade 3: Lymphopenia 7% median follow-up: 14,1 mo OR: 16/44 (36,4%) (15/34 SS, 43%)
NCT03902184	Biological: IPH4102 Drug: Gemcitabine + Oxaliplatin	IPH4102 Alone or in Combination With Chemotherapy in Patients With Advanced T Cell Lymphoma	Recruiting	2	250	Not available
NCT02927925	Drug: Daratumumab	A Study to Assess the Clinical Efficacy and Safety of Daratumumab in Participants With Relapsed or Refractory Natural Killer/T-Cell Lymphoma (NKTCL), Nasal Type	Completed	2	32	[207] Median follow up: 3.1 mo (n=14) ORR – 35,7% Clinical benefit rate - 42,9% CR – 0% PR – 35,7% SD – 7,1% PD – 57,1 Grade 3-4 AEs: 56% (neutropenia, thrombocytopenia, hypotension)
NCT04251065	Drug: Daratumumab	A Multicenter Clinical Trial of Daratumumab in Combination With Gemcitabine, Dexamethasone and Cisplatin in Patients With Relapsed/Refractory CD38 Positive PTCL-NOS, Angioimmunoblastic T-cell Lymphoma AITL and Other Nodal Lymphomas of T Follicular Helper Cells Origin	Not yet recruiting	2	35	Not available
NCT02342782	Biological: Yttrium Y 90 Basiliximab Drug: Carmustine Drug: Etoposide Drug: Cytarabine Drug: Melphalan Procedure: Autologous Hematopoietic Stem Cell Transplantation Other: Laboratory Biomarker Analysis Other: Pharmacological Study	Yttrium Y 90 Basiliximab and Combination Chemotherapy Before Stem Cell Transplant in Treating Patients With Mature T-cell Non-Hodgkin Lymphoma	Active, not recruiting	1	20	Not available
NCT04337593	Drug: Basiliximab Drug: Pegaspargase	Combination of Basiliximab and Pegaspargase in the Treatment of ENKTCL	Not yet recruiting	2	20	Not available
NCT02432235	Drug: ADCT-301	Study of ADCT-301 in Patients With Relapsed or Refractory Hodgkin and Non-Hodgkin Lymphoma	completed	1	133	[208] 39 pts NHL (including CTCL - 8, ATLL - 5, PTCL - 3, AILT -1) TEAEs: 97,4% TEAEs grade ≥3: 74,4% TEAEs leading to discontinuation: 12,8% MTD not reached At doses 60-150 µg/kg ORR: 38,5%, CR: 11,5% TCL pts: ORR 50% (dose ≥60 µg/kg)
NCT04052997	Drug: Camidanlumab Tesirine	Study to Evaluate the Efficacy and Safety of Camidanlumab Tesirine (ADCT-301) in Patients With Relapsed or Refractory Hodgkin Lymphoma	Recruiting	2	100	[209] 47 pts R/R cHL ORR: 80.9% (38/47 pts) CR: with 18 (38.3%) PR: 20 (42.6%) SD: 6 (12.8%) TEAEs: fatigue, nausea, pyrexia, and maculopapular rash, anemia and headache, pruritus, arthralgia, constipation, diarrhea, hypophosphatemia, and rash grade ≥3 TEAEs: 27 (57.4%) pts; the most common (≥5% of pts) hypophosphatemia (6, 12.8%) and gamma-glutamyltransferase increased (3, 6.4%)
NCT02890368	Drug: TTI-621 Monotherapy Drug: TTI-621 + PD-1/PD-L1 Inhibitor Drug: TTI-621 + pegylated interferon-α2a Other: TTI-621 + T-Vec Other: TTI-621 + radiation	Trial of Intratumoral Injections of TTI-621 in Subjects With Relapsed and Refractory Solid Tumors and Mycosis Fungoides	Terminated	1	56	Not available
NCT02663518	Drug: TTI-621 Drug: TTI-621 plus Rituximab Drug: TTI-621 plus Nivolumab	A Trial of TTI-621 for Patients With Hematologic Malignancies and Selected Solid Tumors	Recruiting	1	260	[210] Parts 1−3 (n=214): AEs: infusion-related reaction (IRR, 43%; 3% Gr ≥3), thrombocytopenia (30%; 22% Gr ≥3), chills (21%; 0% Gr ≥3), fatigue (15%; 1% Gr ≥3) Objective responses: - single agent TTI-621: 14/71 (20%) NHL pts: CTCL (n=42, 1 CR, 7 PRs), PTCL (n=22, 2 CRs, 2 PRs) and DLBCL (n=7, 1 CR, 1 PR). Part 4 - 4 dose cohorts (0.5−1.4 mg/kg); 15 pts (MF, n=10; SS n=5) AEs: 11 pts (73%): IRR (n=10), thrombocytopenia (n=3) Gr ≥3 AEs: 4 pts (27%): thrombocytopenia (n=3), IRRs (n=2), exfoliative dermatitis (n=1). 1 PR, 1 skin CR in 6 evaluable pts in the 1 mg/kg cohort 2 responding pts bridged to allogeneic transplantation The mean % change in mSWAT scores -0.4%, -27%, and -37% for 0.5, 0.7 and 1 mg/kg cohorts
NCT03763149	Biological: IBI188	A Study Evaluating the Safety, Tolerability, and Initial Efficacy of Recombinant Human Anti-cluster Differentiation Antigen 47 (CD47) Monoclonal Antibody Injection (IBI188) in Patients With Advanced Malignant Tumors and Lymphomas	Active, not recruiting	1	42	Not available
NCT02520791	Biological: Anti-ICOS Monoclonal Antibody MEDI-570 Other: Laboratory Biomarker Analysis Other: Pharmacological Study	Anti-ICOS Monoclonal Antibody MEDI-570 in Treating Patients With Relapsed or Refractory Peripheral T-cell Lymphoma Follicular Variant or Angioimmunoblastic T-cell Lymphoma	Recruiting	1	38	[211] Pts: AITL (n= 12), PTCL NOS (n=3), CTCL (n=2) PR: 4, SD: 7 grade 3/4 AEs: decreased CD4+ T-cells, anemia (12%), hypophosphatemia (12%), thrombocytopenia (6%), infusion related reactions (6%). No DLTs reported MTD not established
NCT00069238	Biological: Alemtuzumab (Campath) Drug: EPOCH	Campath-1H and EPOCH to Treat Non-Hodgkin's T- and NK-Cell Lymphomas	Active, not recruiting	2	31	Overall Number of Participants Analyzed: 31 CR: 17 (54.8%) PR: 7 (22.6%) PD: 2 (6.5%) SD: 1 (3.2%) Not Evaluable: 4 (12.9%) All-cause mortality: 23/31 (74.19%) Serious AE: 23/31 (74.19%) Other AE: 31/31 (100.00%)
NCT02689453	Biological: IL-15 plus alemtuzumab	Subcutaneous Recombinant Human IL-15 (s.c. rhIL-15) and Alemtuzumab for People With Refractory or Relapsed Chronic and Acute Adult T-cell Leukemia (ATL)	Active, not recruiting	1	10	[212] 11 pts: 7 acute ATL, 2 chronic ATL, 2 PTCL-NOS MTD 2μg/kg/day ORR: 45%, CR: 2/11, PR: 3/11 Hematologic AEs: lymphopenia (11), neutropenia (8), anemia (10), and thrombocytopenia (4). non-hematologic AEs: infusion-related reactions, urticaria
NCT01221571	Drug: AFM 13	A Study to Assess AFM13 in Patients With Hodgkin Lymphoma	Completed	1	28	[126] 28 pts HL doses of 0.01 to 7 mg/kg body weight MTD not reached AEs: mild to moderate PR: 11.5%, SD: 50% Overall disease control rate: 61.5%
NCT03192202	Drug: AFM13	AFM13 in Relapsed/Refractory Cutaneous Lymphomas	Completed	1 2	18	[213] Pts 9 3 treatment cohorts: I-1.5 mg/kg IV weekly, II-7 mg/kg IV weekly, III-7 mg/kg continuous intravenous infusion over 5 days weekly ORR: 50% Cohort I: 1 CR, 1 PR, 1 SD Cohort II: 3 SD Cohort III: 2 PR AE: infusion related reaction (IRR)
NCT04074746	Biological: Anti-CD30/CD16A Monoclonal Antibody AFM13 Drug: Cyclophosphamide Drug: Fludarabine Drug: Fludarabine Phosphate Biological: Genetically Engineered Lymphocyte Therapy	Bispecific Antibody AFM13 Combined With NK Cells for Patients With Recurrent or Refractory CD30 Positive Hodgkin or Non-Hodgkin Lymphomas	Recruiting	1	30	Not available
NCT04101331	Drug: AFM13	II Study to Assess AFM13 in Patients With R/R CD30-positive T-cell Lymphoma or Transformed Mycosis Fungoides	Recruiting	2	145	Not available
NCT04162340	Biological: CD4 CAR T cells	CD4-specific CAR T Cells (CD4 CAR T Cells) for Relapsed/Refractory T Cell Malignancies	Recruiting	1	12	Not available
NCT04219319	Drug: Efficacy of LCAR-T2C CAR-T cells	LCAR-T2C CAR-T Cells in Relapsed or Refractory CD4+ T-cell Lymphoma	Recruiting	1	34	Not available
NCT03081910	Genetic: CD5.CAR/28zeta CAR T cells Drug: Fludarabine Drug: Cytoxan	1 Therapy of Manufactured Autologous T-Cells Expressing a Second Generation Chimeric Antigen Receptor (CAR) for Treatment of T-Cell Malignancies Expressing CD5 Antigen	Recruiting	1	21	Not available
NCT03829540	Biological: CD4CAR	CD4CAR for CD4+ Leukemia and Lymphoma	Recruiting	1	20	Not available
NCT03590574	Biological: AUTO4	I/II Study Evaluating AUTO4 in Patients With TRBC1 Positive T Cell Lymphoma	Recruiting	1 2	55	Not available
NCT01871727	Drug: E7777 9 mcg/kg	A Trial of E7777 in Persistent and Recurrent Cutaneous T-Cell Lymphoma	Recruiting	3	115	Not available
NCT02580552	Drug: Cobomarsen	Safety, Tolerability and Pharmacokinetics of MRG-106 in Patients With Mycosis Fungoides (MF), CLL, DLBCL or ATLL	Active, not recruiting	1	75	[214] Pts 24 I – subcutaneous (SC) injection, II - 2-hour intravenous (IV) infusion III – IV rapid bolus injection (≤ 900 mg/dose) MTD not reached AEs: mostly grade 1-2 Improvement in individually treated lesion or total skin diseases: 23/24 95% (CAILS and mSWAT assessments)
NCT03713320	Drug: Cobomarsen Drug: Vorinostat	SOLAR: Efficacy and Safety of Cobomarsen (MRG-106) vs. Active Comparator in Subjects With Mycosis Fungoides	Active, not recruiting	2	126	Not available
NCT03837457	Drug: Cobomarsen	PRISM: Efficacy and Safety of Cobomarsen (MRG-106) in Subjects With Mycosis Fungoides Who Have Completed the SOLAR Study	Enrolling by invitation	2	60	Not available
NCT01476657	Drug: IPI-145 (duvelisib)	A 1 Study of Duvelisib in Patients With Advanced Hematologic Malignancies	Terminated	1	210	[215] iNHL: ORR: 58% (n = 31), 6 CR relapsed/refractory CLL: 56% (n = 55), 1 CR; peripheral TCL: 50% (n = 16), 3 CR cutaneous TCL, 32% (n = 19). Median time to response: ∼1.8 months. Severe (grade ≥3) AEs: 84% pts neutropenia (32%), alanine transaminase increase (20%), aspartate transaminase increase (15%), anemia and thrombocytopenia (each 14%), diarrhea (11%), and pneumonia (10%) [216] PTCL (n = 16) and CTCL (n = 19), ORR: 50.0% (PTCL) and 31.6% (CTCL) CR: 3 PTCL. grade 3 and 4 AEs: transaminase increases (40% alanine aminotransferase, 17% aspartate aminotransferase), maculopapular rash (17%), neutropenia (17%)
NCT03711578	Drug: Tenalisib,	Efficacy and Safety Study of Tenalisib (RP6530), a Novel PI3K δ/γ Dual Inhibitor in Patients With Relapsed/Refractory Indolent Non-Hodgkin's Lymphoma (iNHL)	Completed	2	20	Not available
NCT03707847	Drug: Crizotinib Drug: Etoposide Capsule Procedure: Auto-HSCT	Crizotinib Combined With Etoposide Capsule Followed by Auto-HSCT for Relapsed and Refractory ALK+ ALCL	Recruiting	4	20	Not available
NCT04439266	Drug: Crizotinib	Testing Crizotinib as a Potential Targeted Treatment in Cancers With ALK Genetic Changes (MATCH-Subprotocol F)	Active, not recruiting	2	35	Not available
NCT02034981	Drug: Crizotinib	2 Study Assessing Efficacy and Safety of Crizotinib in Patients Harboring an Alteration on ALK, MET or ROS1	Active, not recruiting	2	246	Not available
NCT04439253	Drug: Crizotinib	Testing Crizotinib as a Potential Targeted Treatment in Cancers With ROS1 Genetic Changes (MATCH-Subprotocol G)	Active, not recruiting	2	35	Not available
NCT03505554	Drug: Lorlatinib	A Study of Oral Lorlatinib in Patients With Relapsed ALK Positive Lymphoma	Recruiting	2	12	Not available
NCT02419287	Drug: crizotinib	Pilot Study of Crizotinib in Relapsed ALK+ Lymphomas	Unknown status	2	12	Not available
NCT01979536	Drug: Brentuximab Vedotin Drug: Crizotinib Drug: Cyclophosphamide Drug: Cytarabine Drug: Dexamethasone Drug: Doxorubicin Hydrochloride Drug: Etoposide Drug: Ifosfamide Drug: Methotrexate	Brentuximab Vedotin or Crizotinib and Combination Chemotherapy in Treating Patients With Newly Diagnosed Stage II-IV Anaplastic Large Cell Lymphoma	Suspended	2	140	[217] Arm BV of ANHL12P1: addition of brentunixmab vedotin to standard chemotherapy does not cause significantly added toxicity
NCT03116659	Drug: Doxycycline Drug: Imiquimod	CTCL Directed Therapy	Recruiting	Early 2	8	Not available
NCT02341209	Drug: Doxycycline monohydrate	Doxycycline for the Treatment of Cutaneous T-Cell Lymphoma	Recruiting	2	20	Not available

Pts—patients; TCL—T cell lymphoma; BCL—B cell lymphoma, CTCL—cutaneous T cell lymphoma; ATLL—adult T cell leukemia/lymphoma, PTCL—peripheral T cell lymphoma, AILT—angioimmunoblastic T cell lymphoma, ATL—adult T cell leukemiia, PTCL—NOS-peripheral T cell lymphoma not otherwise specified, MTD—maximum tolerated dose; ORR—overall response rate; CR—complete response; CRu—CR unconfirmed; PR—partial response; AE—adverse events; SD—stable disease; PD—progressive disease; TEAES—Treatment Emergent Adverse Events; mo—months

## Conclusion

In contrast to tremendous improvement in the treatment of B cell lymphomas, advances in T cell lymphomas have been hindered by the rarity of each individual subtype, an incomplete understanding of the pathophysiology, and a lack of large clinical trials. Recent fundamental insights into the pathophysiology of TCL have generated potentially ground-breaking therapeutic breakthroughs and resulted in numerous ongoing clinical trials with a variety of target-specific agents. Although therapeutic improvements in TCL, except ALK + ALCL, are not yet spectacular, it might be expected that in the near future, more effective TCL subtype-specific treatments will be elaborated.

## Data Availability

Not applicable.
